# Hierarchical Ensemble Methods for Protein Function Prediction

**DOI:** 10.1155/2014/901419

**Published:** 2014-05-04

**Authors:** Giorgio Valentini

**Affiliations:** AnacletoLab-Dipartimento di Informatica, Università degli Studi di Milano, Via Comelico 39, 20135 Milano, Italy

## Abstract

Protein function prediction is a complex multiclass multilabel classification problem, characterized by multiple issues such as the incompleteness of the available annotations, the integration of multiple sources of high dimensional biomolecular data, the unbalance of several functional classes, and the difficulty of univocally determining negative examples. Moreover, the hierarchical relationships between functional classes that characterize both the Gene Ontology and FunCat taxonomies motivate the development of hierarchy-aware prediction methods that showed significantly better performances than hierarchical-unaware “flat” prediction methods. In this paper, we provide a comprehensive review of hierarchical methods for protein function prediction based on ensembles of learning machines. According to this general approach, a separate learning machine is trained to learn a specific functional term and then the resulting predictions are assembled in a “consensus” ensemble decision, taking into account the hierarchical relationships between classes. The main hierarchical ensemble methods proposed in the literature are discussed in the context of existing computational methods for protein function prediction, highlighting their characteristics, advantages, and limitations. Open problems of this exciting research area of computational biology are finally considered, outlining novel perspectives for future research.

## 1. Introduction

Exploiting the wealth of biomolecular data accumulated by novel high-throughput biotechnologies, “in silico” protein function prediction can generate hypotheses to drive the biological discovery and validation of protein functions [1]. Indeed, “in vitro” methods are costly in time and money, and automatic prediction methods can support the biologist in understanding the role of a protein or of a biological process or in annotating a new genome at high level of accuracy or more, in general, in solving problems of functional genomics [2].

The* Automated Function Prediction* (AFP) is a multiclass, multilabel classification problem characterized by hundreds or thousands of functional classes structured according to a predefined hierarchy. Even in principle, also unsupervised methods can be applied to AFP, due to the inherent difficulty of extracting functional classes without exploiting any available a priori information [3, 4]; usually supervised or semisupervised learning methods are applied in order to exploit the available a priori information about gene annotations.

From a computational standpoint, AFP is a challenging problem for several reasons.

- *The number of functional classes is usually large*: hundreds for the Functional Catalogue (FunCat) [5] or thousands for the Gene Ontology (GO) [6].
- *Proteins may be annotated for multiple functional classes*: since each protein may belong to more than one class at the same time, the classification problem is multilabel.
- *Multiple sources of data are available for each protein*: high-throughput biotechnologies make an increasing number of sources of genomic and proteomic data available. Hence, in order to exploit all the information available for each protein, we need to learn methods that are able to integrate different data sources [7].
- *Functional classes are hierarchically related*: annotations are not independent because functional classes are hierarchically organized; in general, known functional relationships (such as taxonomies) can be exploited to incorporate a priori knowledge in learning algorithms or to introduce explicit constraints between labels.
- *Small number of annotations for each class*: typically, functional classes are severely unbalanced, with a small number of available “positive” annotations.
- *Multiple possible definitions of negative examples*: since we only have positive annotations (the total number of GO negative annotations is about 2500, considering all species (August 2013)), the notion of negative example is not uniquely determined, and different strategies of choosing negative examples can be applied in principle [8].
- *Different reliability of functional labels*: functional annotations have different degrees of evidence; that is, each label is assigned to a gene with a specific level of reliability.
- *Complex and noisy data*: data are usually complex (e.g., high-dimensional, large-scale, and graph-structured) and noisy.

Most of the computational methods for AFP have been applied to unicellular organisms (e.g.,* S. cerevisiae*) [9–11], but recently several approaches have been applied to multicellular organisms (such as* M. musculus* or the* A. thaliana* plant model organisms [2, 12–16]).

Several computational approaches, and in particular machine learning methods, have been proposed to deal with the above issues, ranging from sequence-based methods [17] to network-based methods [18], structured output algorithm based on kernels [19], and hierarchical ensemble methods [20].

Other approaches focused primarily on the integration of multiple sources of data, since each type of genomic data captures only some aspects of the genes to be classified, and a specific source can be useful to learn a specific functional class while being irrelevant to others. In the literature, many approaches have been proposed to deal with this topic, for example, functional linkage networks integration [21], kernel fusion [11], vector space integration [22], and ensemble systems [23].

Extensive experimental studies showed that flat prediction, that is, predictions for each class made independently of the other classes, introduces significant inconsistencies in the classification, due to the violation of the* true path rule* that governs the functional annotations of genes both in the GO and in FunCat taxonomies [24]. According to this rule, positive predictions for a given term must be transferred to its “ancestor” terms and negative predictions to its descendants (see Appendix A and Section 7 for more details about the GO and the true path rule). Moreover flat predictions are difficult to interpret because they may be inconsistent with one another. A method that claims, for example, that a protein has homodimerization activity but does not have dimerization activity is clearly incorrect, and a biologist attempting to interpret these results would not likely trust either prediction [24].

It is worth noting that the results of the Critical Assessment of Functional Annotation (CAFA) challenge, a recent comprehensive critical assessment and comparison of different computational methods for AFP [16], showed that AFP is characterized by multiple complex issues, and one of the best performing CAFA methods corrected flat predictions taking into account the hierarchical relationships between functional terms, with an approach similar to that adopted by hierarchical ensemble methods [25]. Indeed, hierarchical ensemble methods embed in the learning process the relationships between functional classes. Usually, this is performed in a second “reconciliation” step, where the predictions are modified to make them consistent with the ontology [26–29]. More, in general, these methods exploit the relationships between ontology terms, structured according to a forest of trees [5] or a directed acyclic graph [6] to significantly improve prediction performances with respect to “flat” prediction methods [30–32].

Hierarchical classification and in particular ensemble methods for hierarchical classification have been applied in several domains different from protein function prediction, ranging from text categorization [33–35] to music genre classification [36–38], hierarchical image classification [39, 40] and video annotation [41], and automatic classification of worldwide web documents [42, 43]. The present review focuses on hierarchical ensemble methods for AFP. For a more general review on hierarchical classification methods and their applications in different domains, see [44].

The paper is structured as follows. In Section 2, we provide a synthetic picture of the main categories of protein function methods, to properly position hierarchical ensemble methods in the context of computational methods for AFP. In Section 3, the main common characteristics of hierarchical ensemble algorithms, as well as a general taxonomy of these methods, are proposed. The following five sections focus on the main families of hierarchical methods for AFP and discuss their main characteristics.  Section 4 introduces hierarchical top-down methods, Section 5 Bayesian ensemble approaches, Section 6 reconciliation methods, Section 7 true path rule ensemble methods, and the last one (Section 8) ensembles based on decision trees.  Section 9 critically discusses the main issues and limitations of hierarchical ensemble methods and shows that this approach, such as the other current approaches for AFP, cannot be successfully applied without considering the large set of complex learning issues that characterize the AFP problem. The last two sections discuss the open problems and future possible research lines in the context of hierarchical ensemble methods and summarize the main findings in this exciting research area. In the Appendix, some basic information about the FunCat and the GO, that is, the two main hierarchical ontologies that are widely used to annotate proteins in all organisms, are provided, as well as the characteristics of the hierarchical-aware performance measures proposed in the literature to assess the accuracy and the reliability of the predictions made by hierarchical computational methods.

## 2. A Taxonomy of Protein Function Prediction Methods

Several computational methods for the AFP problem have been proposed in the literature. Some methods provided predictions of a relatively small set of functional classes [11, 45, 46], while others considered predictions extended to larger sets, using support vector machines and semidefinite programming [11], artificial neural networks [47], functional linkage networks [21, 48], Bayesian networks [45], or methods that combine functional linkage networks with learning machines using a logistic regression model [12] or simple algebraic operators [13].

Other research lines for AFP explicitly take into account the hierarchical nature of the multilabel classification problem. For instance, structured output methods are based on the joint kernelization of both input variables and output labels, using, for example, perceptron-like learning algorithms [49] or maximum-margin algorithms [50]. Other approaches improve the prediction of GO annotations by extracting implicit semantic relationships between genes and functions [51]. Finally, other methods adopted an ensemble approach [52] to take advantage of the intrinsic hierarchical nature of protein function prediction, explicitly considering the relationships between functional classes [24, 53–55].

Computational methods for AFP, mostly based on machine learning methods, can be schematically grouped in the following four families:

1. sequence-based methods;
2. network-based methods;
3. kernel methods for structured output spaces;
4. hierarchical ensemble methods.

This grouping is neither exhaustive nor strict, meaning that certain methods do not belong to any of these groups, and others belong to more than one.

### 2.1. Sequence-Based Methods

Algorithms based on alignment of sequences represent the first attempts to computationally predict the function of proteins [56, 57]: similar sequences are likely to share common functions, even if it is well known that secondary and tertiary structure conservation are usually more strictly related to protein functions. However, algorithms able to infer similarities between sequences are today standard methods of assigning functions to proteins in newly sequenced organisms [17, 58]. Of course, global or local structure comparison algorithms between proteins can be applied to detect functional properties [59], and, in this context, the integration of different sequence and structure-based prediction methods represents a major challenge [60].

Even if most of the research efforts for the design and development of AFP methods concentrated on machine learning methods, it is worth noting that in the AFP 2011 challenge [16] one of the best performing methods is represented by a sequence-based algorithm [61]. Indeed, when the only information available is represented by a raw sequence of amino acids or nucleotides, sequence-based methods can be competitive with state-of-the-art machine learning methods by exploiting homology-based inference [62].

### 2.2. Network-Based Methods

These methods usually represent each dataset through an undirected graph *G* = (*V*, *E*), where nodes *v* ∈ *V* correspond to gene/gene products and edges *e* ∈ *E* are weighted according to the evidence of cofunctionality implied by data source [63, 64]. These algorithms are able to transfer annotations from previously annotated (labeled) nodes to unannotated (unlabeled) ones by exploiting “proximity relationships” between connected nodes. Basically, these methods are based on transductive label propagation algorithms that predict the labels of unannotated examples without using a global predictive model [14, 21, 45]. Several method exploited the semantic similarity between GO terms [65, 66] to derive functional similarity measures between genes to construct functional terms, using then supervised or semisupervised learning algorithm to infer GO annotations of genes [67–70].

Different strategies to learn the unlabeled nodes have been explored by “label propagation” algorithms, that is, methods able to “propagate” the labels of annotated proteins across the networks, by exploiting the topology of the underlying graph. For instance, methods based on the evaluation of the functional flow in graphs [64, 71], methods based on the Hopfield networks [48, 72, 73], methods based on the Markov [74, 75] and Gaussian random fields [14, 46], and also simple “guilt-by-association” methods [76, 77], based on the assumption that connected nodes/proteins in the functional networks are likely to share the same functions. Recently, methods based on kernelized score functions, able to exploit both local and global semisupervised learning strategies, have been successfully applied to AFP [78] as well as to disease gene prioritization [79] and drug repositioning problems [80, 81].

Reference [82] showed that different graph-based algorithms can be cast into a common framework where a quadratic cost objective function is minimized. In this framework, closed form solutions can be derived by solving a linear system of size equal to the cardinality of nodes (proteins) or using fast iterative procedures such as the Jacobi method [83]. A network-based approach, alternative to label propagation and exhibiting strong theoretical predictive guarantees in the so-called mistake bound model, has been recently proposed by [84].

### 2.3. Kernel Methods for Structured Output Spaces

By extending kernels to the output space, the multilabel hierarchical classification problem is solved globally: the multilabels are viewed as elements of a structured space modeled by suitable kernel functions [85–87], and structured predictions are viewed as a maximum a posteriori prediction problem [88].

Given a feature space *𝒳* and a space of structured labels *𝒴*, the task is to learn a mapping *f* : *𝒳* → *𝒴* by an induced joint kernel function *k* that computes the “compatibility” of a given input-output pair (*x*, *y*): for each test example *x* ∈ *𝒳*, we need to determine the label $\bar{y}\in 𝒴$ such that $\bar{y}=\arg\max_{y\in 𝒴}k(x,y)$, for any *x* ∈ *𝒳*. By modeling probabilities by a log-linear model, and using a suitable feature map *ϕ*(*x*, *y*), we can define an induced joint kernel function that uses both inputs and outputs to compute the “compatibility” of a given input-output pair [88]

(1) $$\begin{array}{c} k:\left(𝒳\times 𝒴\right)\times \left(𝒳\times 𝒴\right)⟶\mathbb{R}. \end{array}$$

Structured output methods infer a label $\hat{y}$ by finding the maximum of a function *g* that uses the previously defined joint kernel (1)

(2) $$\begin{array}{c} \hat{y}=\arg\underset{y\in 𝒴}{\max\;}g\left(x,y\right). \end{array}$$

The* GOstruct* system implemented a structured perceptron and a variant of the structured support vector machine [85]. This approach has been successfully applied to the prediction of GO terms in mouse and other model organisms [19]. Structured output maximum-margin algorithms have been also applied to the tree-structured prediction of enzyme functions [50, 86].

### 2.4. Hierarchical Ensemble Methods

Other approaches take explicitly into account the hierarchical relationships between functional terms [26, 29, 53, 54, 89, 90]. Usually, they modify the “flat” predictions (i.e., predictions made independently of the hierarchical structure of the classes) and correct them improving accuracy and consistency of the multilabel annotations of proteins [24].

The flat approach makes predictions for each term independently and, consequently, the predictor may assign to a single protein a set of terms that are inconsistent with one another. A possible solution for this problem is to train a classifier for each term of the reference ontology to produce a set of prediction at each term and, finally, to reconcile the predictions by taking into account the relationships between the classes of the ontology. Different ensemble based algorithms have been proposed ranging from methods restricted to multilabels with single and no partial paths [91] to methods extended to multiple and also partial paths [92]. Many recent published works clearly demonstrated that this approach ensures an increment in precision, but this comes at expenses of the overall recall [2, 30].

In the next section, we discuss in detail hierarchical ensemble methods, since they constitute the main topic of this review.

## 3. Hierarchical Ensemble Methods: Exploiting the Hierarchy to Improve Protein Function Prediction

**Figure 1:**
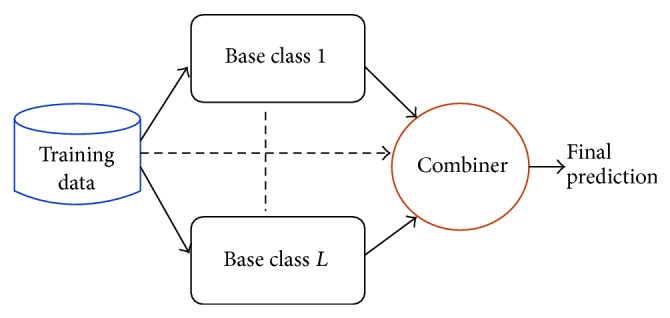
Ensemble of classifiers.

Ensemble methods are one of the main research areas of machine learning [52, 93–95]. From a general standpoint, ensembles of classifiers are sets of learning machines that work together to solve a classification problem (Figure 1). Empirical studies showed that in both classification and regression problems ensembles improve on single learning machines, and, moreover, large experimental studies compared the effectiveness of different ensemble methods on benchmark data sets [96–99], and they have been successfully applied to several computational biology problems [100–104]. Ensemble methods have been also successfully applied in an unsupervised setting [105, 106]. Several theories have been proposed to explain the characteristics and the successful application of ensembles to different application domains. For instance, Allwein, Schapire, and Singer interpreted the improved generalization capabilities of ensembles of learning machines in the framework of large margin classifiers [107, 108]; Kleinberg, in the context of Stochastic Discrimination Theory [109], and Breiman and Friedman in the light of the bias-variance analysis borrowed from classical statistics [110, 111]. The interest in this research area is motivated also by the availability of very fast computers and networks of workstations at a relatively low cost that allow the implementation and the experimentation of complex ensemble methods using off-the-shelf computer platforms.

Constraints between labels and, more in general, the issue of label dependence have been recognized to play a central role in multilabel learning [112]. Protein function prediction can be regarded as a paradigmatic multilabel classification problem, where the exploitation of a priori knowledge about the hierarchical relationships between the labels can dramatically improve classification performance [24, 27, 113].

In the context of AFP problems, ensemble methods reflect the hierarchy of functional terms in the structure of the ensemble itself: each base learner is associated with a node of the graph representing the functional hierarchy and learns a specific GO term or FunCat category. The predictions provided by the trained classifiers are then combined by exploiting the hierarchical relationships of the taxonomy.

In their more general form, hierarchical ensemble methods adopt a two-step learning strategy.

1. In the first step, each base learner separately or interacting with connected base learners learns the protein functional category on a per term basis. In most cases, this yields a set of independent classification problems, where each base learning machine is trained to learn a specific functional term, independently of the other base learners.
2. In the second step, the predictions provided by the trained classifiers are combined by considering the hierarchical relationships between the base classifiers modeled according to the hierarchy of the functional classes.

**Figure 2:**
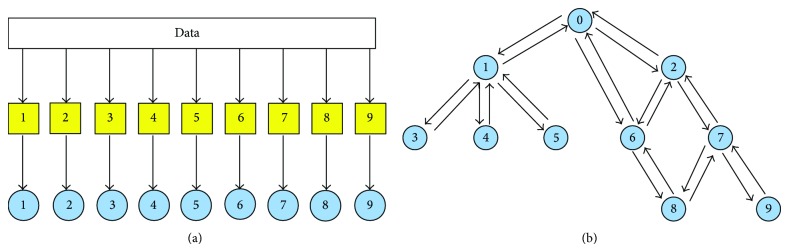
Schematic representation of the two main learning steps of hierarchical ensemble methods. (a) Training of base classifiers; (b) top-down and/or bottom-up propagation of the predictions.

Figure 2 depicts the two learning steps of hierarchical ensemble methods. In the first step, a learning algorithm (a square object in Figure 2(a)) is applied to train the base classifiers associated with each class (represented with numbers from 1 to 9). Then, the resulting base classifiers (circles) in the prediction phase exploit the hierarchical relationships between classes to combine its predictions with those provided by the other base classifiers (Figure 2(b)). Note that the dummy 0 node is added to obtain a rooted hierarchy. Up and down arrows represent the possibility of combining predictions by exploiting those provided, respectively, by children and parents classifiers, according to a bottom-up or top-down learning strategy. Note that both “local” combinations are possible (e.g., the prediction of node 5 may depend only on the prediction of node 1), but also “global” combinations can be considered, by taking into account the predictions across the overall structure of the graph (e.g., predictions for node 9 can depend on all the predictions made by all the other base classifiers from 1 to 8). Moreover, both top-down propagation of the predictions (down arrows, Figure 2(b)) and bottom-up propagation (up arrows) can be considered, depending on the specific design of the hierarchical ensemble algorithm.

This ensemble approach is highly modular: in principle, any learning algorithm can be used to train the classifiers in the first step, and both annotation decisions, probabilities, or whatever scores provided by each base learner can be combined, depending on the characteristics of the specific hierarchical ensemble method.

In this section, we provide some basic notations and an ensemble taxonomy that will be used to introduce the different hierarchical ensemble methods for AFP.

### 3.1. Basic Notation

A gene/gene product *g* can be represented through a vector **x** ∈ ℝ^*d*^ having *d* different features (e.g., gene expression levels across *d* different conditions, sequence similarities with other genes/proteins, or presence or absence of a given domain in the corresponding protein or genetic or physical interaction with other proteins). Note that we, for the sake of simplicity and with a certain approximation, refer in the same way to genes and proteins, even if it is well known that a given gene may correspond to multiple proteins. A gene *g* is assigned to one or more functional classes in the set *C* = {*c*_1_, *c*_2_,…, *c*_*m*_} structured according to a FunCat forest of trees *T* or a directed acyclic graph *G* of the Gene Ontology (usually a dummy root class *c*_0_, which every gene belongs to, is added to *T* or *G* to facilitate the processing). The assignments are coded through a vector of multilabels **y** = (*y*_1_, *y*_2_,…, *y*_*m*_)∈{0,1}^*m*^, where *g* belongs to class *c*_*i*_ if and only if *y*_*i*_ = 1.

In both the Gene Ontology(GO) and FunCat taxonomies, the functional classes are structured according to a hierarchy and can be represented by a directed graph, where nodes correspond to classes and edges correspond to relationships between classes. Hence, the node corresponding to the class *c*_*i*_ can be simply denoted by *i*. We represent the set of children nodes of *i* by child(*i*), and the set of its parents by par(*i*). Moreover, *y*_child(*i*)_ denotes the labels of the children classes of node *i* and analogously *y*_par(*i*)_ denotes the labels of the parent classes of *i*. Note that in FunCat only one parent is permitted, since the overall hierarchy is a tree forest, while, in the GO, more parents are allowed, because the relationships are structured according to a directed acyclic graph.

Hierarchical ensemble methods train a set of calibrated classifiers, one for each node of the taxonomy *T*. These classifiers are used to derive estimates ${\hat{p}}_{i}(g)$ of the probabilities *p*_*i*_(*g*) = *ℙ*(*V*_*i*_ = 1∣*V*_par(*i*)_ = 1, *g*) for all *g* and *i*, where (*V*_1_,…, *V*_*m*_)∈{0,1}^*m*^ is the vector random variable modeling the unknown multilabel of a gene *g*, and *V*_par(*i*)_ denotes the random variables associated with the parents of node *i*. Note that *p*_*i*_(*g*) are probabilities conditioned to *V*_par(*i*)_ = 1, that is, the probability that a gene is annotated to a given term *i*, given that the gene is just annotated to its parent terms, thus respecting the true path rule. Ensemble methods infer a multilabel assignment $\hat{y}=({\hat{y}}_{1},\ldots ,{\hat{y}}_{m})\in {\left\{0,1\right\}}^{m}$ based on estimates ${\hat{p}}_{1}(g),\ldots ,{\hat{p}}_{m}(g)$.

### 3.2. A Taxonomy of Hierarchical Ensemble Methods

Hierarchical ensemble methods for AFP share several characteristics, from the two-step learning approach to the exploitation of the hierarchical relationships between classes. For these reasons, it is quite difficult to clearly and univocally individuate taxonomy of hierarchical ensemble methods. Here, we show taxonomy useful mainly to describe and discuss existing methods for AFP. For a recent review and taxonomy of hierarchical ensemble methods, not specific for AFP problems, we refer the reader to the comprehensive Silla and others' review [44].

In the following sections, we discuss the following groups of hierarchical ensemble methods:

- top-down ensemble methods. These methods are characterized by a simple top-down approach in the second step: only the output of the parent node/base classifier influences the output of the children, thus resulting in a top-down propagation of the decisions;
- Bayesian ensemble methods. These are a class of methods theoretically well founded and in some cases they are optimal from a Bayesian standpoint;
- Reconciliation methods. This is a heterogeneous class of heuristics by which we can combine the predictions of the base learners, by adopting different “local” or “global” combination strategies;
- true path rule ensembles. These methods adopt a heuristic approach based on the “true path rule” that governs both the GO and FunCat ontologies;
- decision tree-based ensembles. These methods are characterized by the application of decision trees as base learners or by adopting decision tree-like learning strategies to combine predictions of the base learners.

Despite this general characterization, several methods could be assigned to different groups, and for several hierarchical ensemble methods it is difficult to assign them to any the introduced classes of methods.

For instance, in [114–116] the authors used the hierarchy only to construct training sets different for each term of the Gene Ontology, by determining positive and negative examples on the basis of the relationships between functional terms. In [89] for each classifier associated with a node, a gene is labeled as positive (i.e., belonging to the term associated with that node) if it actually belongs to that node or as negative if it does not belong to that node or to the ancestors or descendants of the node.

Other approaches exploited the correlation between nearby classes [32, 53, 117]. Shahbaba and Neal [53] take into account the hierarchy to introduce correlation between functional classes, using a multinomial logit model with Bayesian priors in the context of* E. coli* functional classification with Riley's hierarchies [118]. Bogdanov and Singh incorporated functional interrelationships between terms during the extraction of features based on annotations of neighboring genes and then applied a nearest-neighbor classifier to predict protein functions [117]. The HiBLADE method (hierarchical multilabel boosting with label dependency) [32] not only takes advantage of the preestablished hierarchical taxonomy of the classes but also effectively exploits the hidden correlation among the classes that is not shown through the class hierarchy, thereby improving the quality of the predictions. In particular, the dependencies of the children for each label in the hierarchy are captured and analyzed using the Bayes method and instance-based similarity. Experiments using the FunCat taxonomy and the yeast model organism show that the proposed method is competitive with TPR-W (Section 7.2) and HABYES-CS (Section 5.3) hierarchical ensemble methods.

An adaptation of a classical multiclass boosting algorithm [119] has been adapted to fit the hierarchical structure of the FunCat taxonomy [120]: the method is relatively simple and straightforward to be implemented and achieves competitive results for the AFP in the yeast model organism.

Finally, other hierarchical approaches have been proposed in the context of competitive networks learning framework. Competitive networks are well-known unsupervised and supervised methods able to map the input space into a structured output space where clusters or classes are usually arranged according to a grid topology and where learning adopts at the same way a competition, cooperation, and adaptation strategy [121]. Interestingly enough, in [122], the authors adopted this approach to predict the hierarchy of gene annotations in the yeast model organism, by using a tree-topology according to the FunCat taxonomy: each neuron is connected with its parent or with its children. Moreover, each neuron in tree-structured output layer is connected to all neurons of the input layer, representing the instances, that is, the set of genomic features associated with each gene to be classified. Results obtained with the hierarchy of enzyme commission codes showed that this approach is competitive with those obtained with hierarchical decision trees ensembles [29] (Section 8).

To provide a general picture of the methods discussed in the following sections, Table 1 summarizes their main characteristics. The first two columns report the name and a reference to the method, the third whether multiple or single paths across the taxonomy are allowed, and the next whether partial paths are considered (i.e., paths that do not end with a leaf). The successive columns refer to the class structure (a tree or a DAG), to the adoption or not of cost-sensitive (i.e., unbalance-aware) classification approaches, and to the adoption of strategies to properly select negative examples in the training phase. Finally, the last three columns summarize the type of the base learner used (“spec” means that only a specific type of base learner is allowed and “any” means that any type of learner can be used within the method), whether the method improves or not with respect to the flat approach, and the mode of processing of the nodes (“TD”: top-down approach, and “TD&BUP”: adopting both top-down and bottom-up strategies). Of course methods having more checkmarks are more flexible and in general methods that can process a DAG can also process tree-structured ontologies, but the opposite is not guaranteed, while the type of node processing relies on the way the information is propagated across the ontology. It is worth noting that all the considered methods improve on baseline “flat” classification methods.

## 4. Hierarchical Top-Down (HTD) Ensembles

These ensemble methods exploit the hierarchical relationships between functional terms in a top-to-bottom fashion, that is, considering only the relationships denoted by the down arrows in Figure 2(b). The basic hierarchical top-down ensemble method (HTD) algorithm is straightforward: for each gene *g*, starting from the set of nodes at the first level of the graph *G* (denoted by root(*G*)), the classifier associated with the node *i* ∈ *G* computes whether the gene belongs to the class *c*_*i*_. If yes, the classification process continues recursively on the nodes *j* ∈ child(*i*); otherwise, it stops at node *i*, and the nodes belonging to the descendants rooted at *i* are all set to 0. To introduce the method, we use probabilistic classifiers as base learners trained to predict class *c*_*i*_ associated with the node *i* of the hierarchical taxonomy. Their estimates ${\hat{p}}_{i}(g)$ of *ℙ*(*V*_*i*_ = 1∣*V*_par(*i*)_ = 1, *g*) are used by the HTD ensemble to classify a gene *g* as follows:

(3) $$\begin{array}{c} {\hat{y}}_{i}=\{\begin{array}{ccc} \left\{{\hat{p}}_{i}\left(g\right)>\frac{1}{2}\right\} & \text{if}\;i\in \text{root}\left(G\right) & \\ \left\{{\hat{p}}_{i}\left(g\right)>\frac{1}{2}\right\} & \text{if}\;i\notin \text{root}\left(G\right) & \wedge \left\{{\hat{p}}_{\text{par}(i)}\left(g\right)>\frac{1}{2}\right\}\\ 0 & \text{if}\;i\notin \text{root}\left(G\right) & \wedge \left\{{\hat{p}}_{\text{par}(i)}\left(g\right)\leq \frac{1}{2}\right\}, \end{array} \end{array}$$

where {*x*} = 1 if *x* > 0; otherwise, {*x*} = 0 and ${\hat{p}}_{\text{par}(i)}$ is the probability predicted for the parent of the term *i*. It is easy to see that this procedure ensures that the predicted multilabels $\hat{y}=({\hat{y}}_{1},\ldots ,{\hat{y}}_{m})$ are consistent with the hierarchy. We can apply the same top-down procedure also using nonprobabilistic classifiers, that is, base learners generating continuous scores, or also discrete decisions, by slightly modifying (3).

In [123], a cost-sensitive version of the basic top-down hierarchical ensemble method HTD has been proposed: by assigning ${\hat{y}}_{i}$ before the label of any *j* in the subtree rooted at *i*, the following rule is used:

(4) $$\begin{array}{c} {\hat{y}}_{i}=\left\{{\hat{p}}_{i}\geq \frac{1}{2}\right\}\times \left\{{\hat{y}}_{\text{par}(i)}=1\right\} \end{array}$$

for *i* = 1,…, *m* (note that the guessed label ${\hat{y}}_{0}$ of the root of *G* is always 1). Then, the cost-sensitive variant HTD-CS introduces a single cost-sensitive parameter *τ* > 0 which replaces the threshold 1/2. The resulting rule for HTD-CS is then

(5) $$\begin{array}{c} {\hat{y}}_{i}=\left\{{\hat{p}}_{i}\geq \tau \right\}\times \left\{{\hat{y}}_{\text{par}(i)}=1\right\}. \end{array}$$

By tuning *τ*, we may obtain ensembles with different precision/recall characteristics. Despite the simplicity of the hierarchical top-down methods, several works showed their effectiveness for AFP problems [28, 31].

**Figure 3:**
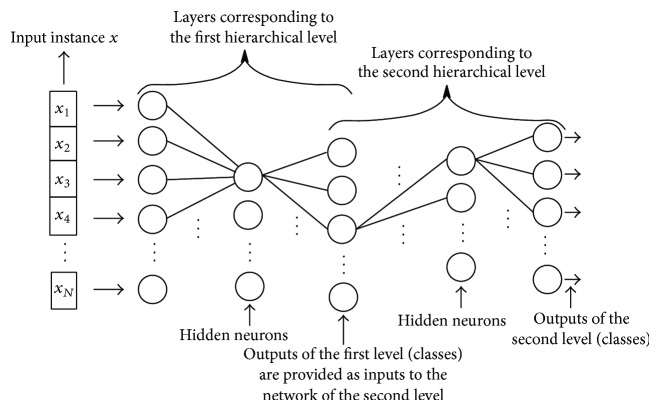
HMC-LMLP: outputs of the MLP responsible for the predictions in the first level are used as input to another MLP for the predictions in the second level (adapted from [125]).

For instance, Cerri and De Carvalho experimented different variants of top-down hierarchical ensemble methods for AFP [28, 124, 125]. The HMC-LMLP (hierarchical multilabel classification with local multilayer perceptron) successively trains a local MLP network for each hierarchical level, using the classical backpropagation algorithm [126]. Then, the output of the MLP for the first layer is used as input to train the MLP that learns the classes of the second level and so on (Figure 3). A gene is annotated to a class if its corresponding output in the MLP is larger than a predefined threshold; then, in a postprocessing phase (second-step of the hierarchical classification), inconsistent predictions are removed (i.e., classes predicted without the prediction of their superclasses) [125]. In practice, instead of using a dichotomic classifier for each node, the HMC-LMLP algorithm applies a single multiclass multilayer perceptron for each level of the hierarchy.

A related approach adopts multiclass classifiers (HTD-MULTI) for each node, instead of a simple binary classifier, and tries to find the most likely path from the root to the leaves of the hierarchy, considering simple techniques, such as the multiplication or the sum of the probabilities estimated at each node along the path [127]. The method has been applied to the cell cycle branch of the FunCat hierarchy with the yeast model organism, showing improvements with respect to classical hierarchical top-down methods, even if the proposed approach can only predict classes along a single “most likely path,” thus not considering that in AFP we may have annotations involving multiple and partial paths.

Another method that introduces multiclass classifiers instead of simple dichotomic classifiers has been proposed by Paes et al. [128]: local per level multiclass classifiers (HTD-PERLEV) are trained to distinguish between the classes of a specific level of the hierarchy, and two different strategies to remove inconsistencies are introduced. The method has been applied to the hierarchical classification of enzymes using the EC taxonomy for the hierarchical classification of enzymes, but unfortunately this algorithm is not well suited to AFP, since leaf nodes are mandatory (that is partial path annotations are not allowed) and multilabel annotations along multiple paths are not allowed.

Another interesting top-down hierarchical approach proposed by the same authors is HMC-LP (hierarchical multilabel classification label-powerset), a hierarchical variation of the* label-powerset* nonhierarchical multilabel method [129], that has been applied to the prediction of gene function of the yeast model organism using 10 different data sets and the FunCat taxonomy [124]. According to the* label-powerset* approach, the method is based on a first label-combination step by which, for each example (gene), all classes assigned to the example are combined into a new and unique class, and this process is repeated for each level of the hierarchy. In this way, the original problem is transformed into a hierarchical single-label problem. In both the training and test phases, the top-down approach is applied, and at the end of the classification phase the original classes can be easily reconstructed [124]. In an experimental comparison using the FunCat taxonomy for* S. cerevisiae,* results showed that hierarchical top-down ensemble methods significantly outperform decision trees-based hierarchical methods, but no significant difference between different flavors of top-down hierarchical ensembles has been detected [28].

Top-down algorithms can be conceived also in the context of network-based methods (HTD-NET). For instance, in [26], a probabilistic model that combines relational protein-protein interaction data and the hierarchical structure of GO to predict true-path consistent function labels obeys the true path rule by setting the descendants of a node as negative whenever that node is set to negative. More precisely, the authors at first compute a local hierarchical conditional probability, in the sense that, for any nonroot GO term, only the parents affect its labeling. This probability is computed within a network-based framework assuming that the labeling of a gene is independent of any other genes given that of its neighbors (a sort of the Markov property with respect to gene functional interaction networks) and assuming also a binomial distribution for the number of neighbors labeled with child terms with respect to those labeled with the parent term. These assumptions are quite stringent but are necessary to make the model tractable. Then, a global hierarchical conditional probability is computed by recursively applying the previously computed local hierarchical conditional probability by considering all the ancestors. More precisely, by assuming that $\mathbb{P}({\hat{y}}_{i}=1|g,N(g))$, that is, the probability that a gene *g* is annotated for a a node *i*, given the status of the annotations of its neighborhood *N*(*g*) in the functional networks, the global hierarchical conditional probability factorizes according to the GO graph as follows:

(6) ℙ(y^i=1 ∣ g,N(g)) =∏j∈anc(i)ℙ(y^j=1 ∣ y^par(j)=1,Nloc⁡(g)),

where *N*_loc⁡_(*g*) represents the local hierarchical neighborhood information on the parent-child GO term pair par(*j*) and *j* [26]. This approach guarantees to produce GO term label assignments consistent with the hierarchy, without the need of a postprocessing step.

Finally in [130], the author applied a hierarchical method to the classification of yeast FunCat categories. Despite its well-founded theoretical properties based on large margin methods, this approach is conceived for one path hierarchical classification, and hence it results to be unsuited for hierarchical AFP, where usually multiple paths in the hierarchy should be considered, since in most cases genes can play different functional roles in the cell.

## 5. Ensemble Based Bayesian Approaches for Hierarchical Classification

These methods introduce a Bayesian approach to the hierarchical classification of proteins, by using the classical Bayes theorem or Bayesian networks to obtain tractable factorizations of the joint conditional probabilities from the original “full Bayesian” setting of the hierarchical AFP problem [20, 30] or to achieve “Bayes-optimal” solutions with respect to loss functions well suited to hierarchical problems [27, 92].

### 5.1. The Solution Based on Bayesian Networks

One of the first approaches addressing the issue of inconsistent predictions in the Gene Ontology is represented by the Bayesian approach proposed in [20] (BAYES NET-ENS). According to the general scheme of hierarchical ensemble methods, two main steps characterize the algorithm:

1. flat prediction of each term/class (possibly inconsistent);
2. Bayesian hierarchical combination scheme to allow collaborative error-correction over all nodes.

After training a set of base classifiers on each of the considered GO terms (in their work, the authors applied the method to 105 selected GO terms), we may have a set of (possibly inconsistent) $\hat{y}$ predictions. The goal consists in finding a set of consistent **y** predictions, by maximizing the following equation derived from the Bayes theorem:

(7) ℙ(y1,…,yn ∣ y^1,…,y^n) =ℙ(y^1,…,y^n ∣ y1,…,yn)ℙ(y1,…,yn)Z,

where *n* is the number of GO nodes/terms and *Z* is a constant normalization factor.

Since the direct solution of (7) is too hard, that is, exponential in time with respect to the number of nodes, the authors proposed a Bayesian network structure to solve this difficult problem, in order to exploit the relationships between the GO terms. More precisely, to reduce the complexity of the problem, the authors imposed the following constraints:

1. *y*_*i*_ nodes conditioned to their children (GO structure constraints);
2. ${\hat{y}}_{i}$ nodes conditioned on their label *y*_*i*_ (the Bayes rule);
3. ${\hat{y}}_{i}$ are independent from both ${\hat{y}}_{j}$, *i* ≠ *j*, and *y*_*j*_, *i* ≠ *j*, given *y*_*i*_.

**Figure 4:**
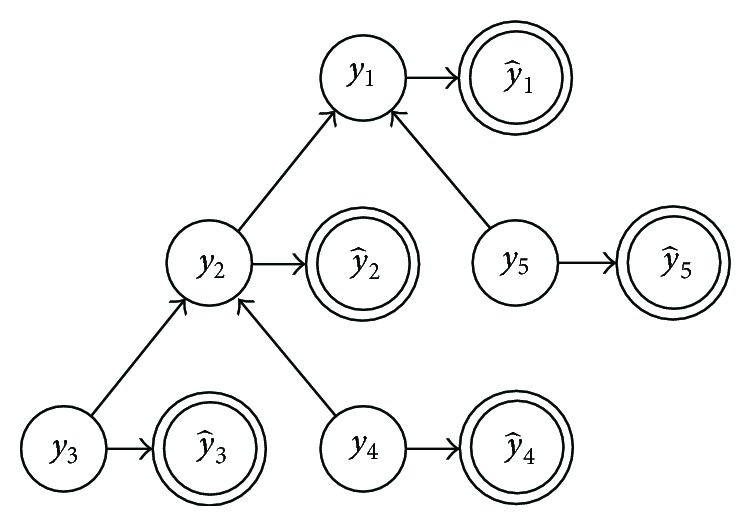
Bayesian network involved in the hierarchical classification (adapted from [20]).

In other words, we can ensure that a label is 1 (positive) when any one of its children is 1 and the edges from *y*_*i*_ to ${\hat{y}}_{i}$ assure that a classifier output ${\hat{y}}_{i}$ is a random variable independent of all other classifier outputs ${\hat{y}}_{j}$ and labels *y*_*j*_, given its true label *y*_*i*_ (Figure 4).

More precisely, from the previous constraints we can derive the following equations:

(8) from  the  first  constraint: ℙ(y1,…,yn)=∏i=1nℙ(yi ∣   child(yi))

(9) from  the  last  two  constraints: ℙ(y^1,…,y^n ∣ y1,…,yn)=∏i=1nℙ(y^i ∣ yi).

**Figure 5:**
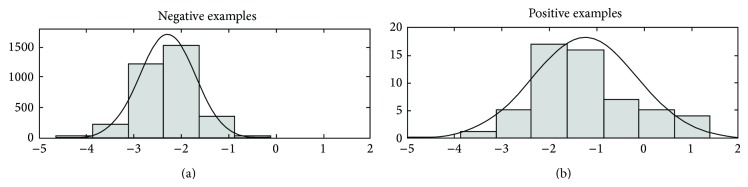
Distribution of positive and negative validation examples (a Gaussian distribution is assumed). Adapted from [20].

Note that (8) can be inferred from training labels simply by counting, while (9) can be inferred by validation during training, by modeling the distribution of ${\hat{y}}_{i}$ outputs over positive and negative examples, by assuming a parametric model (e.g., Gaussian distribution; see Figure 5).

For the implementation of their method, the authors adopted bagged ensemble of SVMs [131] to make their predictions more robust and reliable at each node of the GO hierarchy, and median values of their outputs on out-of-bag examples have been used to estimate means and variances for each class. Finally, means and variances have been used as parameters of the Gaussian models used to estimate the conditional probabilities of (9).

**Figure 6:**
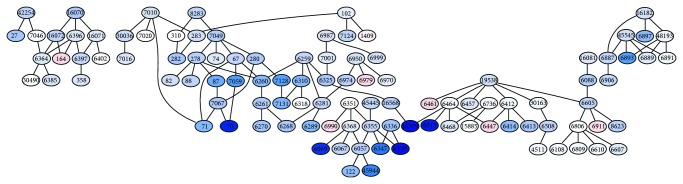
Improvements induced by the hierarchical prediction of the GO terms. Darker shades of blue indicate largest improvements, and darker shades of red indicate largest deterioration; white means no change (adapted from [20]).

Results with the 105 terms/nodes of the GO BP (model organism* S. cerevisiae*) showed substantial improvements with respect to nonhierarchical “flat” predictions: the hierarchical approach improves AUC results on 93 of the 105 GO terms (Figure 6).

### 5.2. The Markov Blanket and Approximated Breadth First Solution

In [30], the authors proposed an alternative approximated solution to the complex equation (7) by introducing the following two variants of the Bayesian integration:

1. HIER-MB: hierarchical Bayesian combination involving nodes in the Markov blanket.
2. HIER-BFS: hierarchical Bayesian combination involving the 30 first nodes visited through a breadth-first-search (BFS) in the GO graph.

The method has been applied to the prediction of more than 2000 GO terms for the mouse model organism and performed among the top methods in the* MouseFunc* challenge [2].

**Figure 7:**
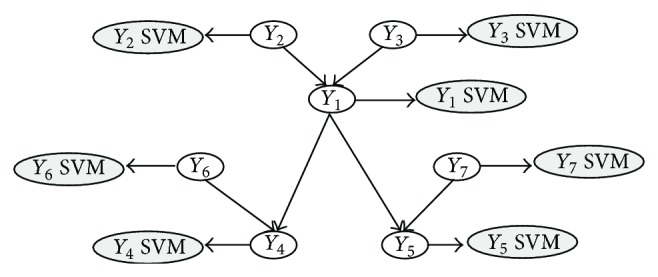
Markov blanket surrounding the GO term *Y*_1_. Each GO term is represented as a blank node, while the SVM classifier output is represented as a gray node (adapted from [30]).

The first approach (HIER-MB) modifies the output of the base learners (SVMs in the Guan et al. paper) taking into account the Bayesian network constructed using the Markov blanket surrounding the GO term of interest (Figure 7). In a Bayesian network, the Markov blanket of a node *i* is represented by its parents (par(*i*)), its children (child(*i*)), and its children's other parents. The Bayesian network involving the Markov blanket of node *i* is used to provide the prediction ${\hat{y}}_{i}$ of the ensemble, thus leveraging the local relationships of node *i* and the predictions for the nodes included in its Markov blanket.

**Figure 8:**
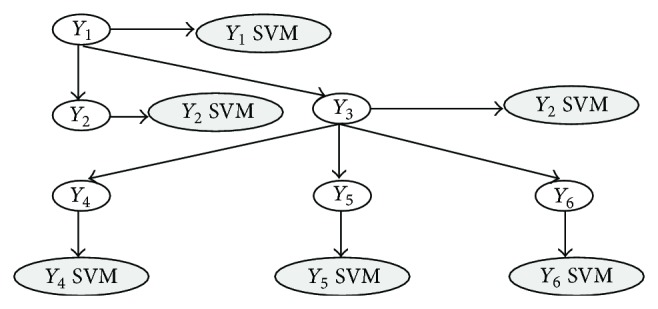
The breadth-first subnetwork stemming from *Y*_1_. Each GO term is represented through a blank node and the SVM outputs are represented as gray nodes (adapted from [30]).

To enlarge the size of the Bayesian subnetwork involved in the prediction of the node of interest, a variant based on the Bayesian networks constructed by applying a classical breadth-first search is the basis of the HIER-BFS algorithm. To reduce the complexity at most, 30 terms are included (i.e., the first 30 nodes reached by the breadth-first algorithm; see Figure 8). In the implementation, ensembles of 25 SVMs have been trained for each node, using vector space integration techniques [132] to integrate multiple sources of data.

Note that with both HIER-MB and HIER-BFS methods we do not take into account the overall topology of the GO network but only the terms related to the node for which we perform the prediction. Even if this general approach is reasonable and achieves good results, its main drawback is represented by the locality of the hierarchical integration (limited to the Markov blanket and the first 30 BFS nodes). Moreover, in previous works, it has been shown that the adopted integration strategy (vector space integration) is in most cases worse than kernel fusion [11] and ensemble methods for data integration [23].

**Figure 9:**
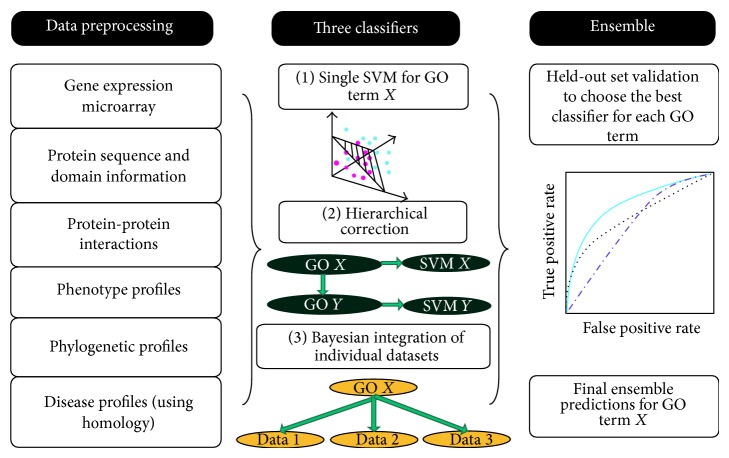
Integration of diverse methods and diverse sources of data in an ensemble framework for AFP prediction. The best classifier for each GO term is selected through held-out set validation (adapted from [30]).

In the same work [30], the authors propose also a sort of “test and select” method [133], by which three different classification approaches (a) single flat SVMs, (b) Bayesian hierarchical correction, and (c) Naive Bayes combination are applied, and for each GO term the best one is selected by internal cross-validation (Figure 9).

It is worth noting that other approaches adopted Bayesian networks to resolve the hierarchical constraints underlying the GO taxonomy. For instance, in the* FALCON* algorithm the GO is modeled as a Bayesian network and for any given input the algorithm returns the most probable GO term assignment in accordance with the GO structure, by using an evolutionary-based optimization algorithm [134].

### 5.3. HBAYES: An “Optimal” Hierarchical Bayesian Ensemble Approach

The HBAYES ensemble method [92, 135] is a general technique for solving hierarchical classification problems on generic taxonomies *G* structured according to forest of trees. The method consists in training a calibrated classifier at each node of the taxonomy. In principle, any algorithm (e.g., support vector machines or artificial neural networks) whose classifications are obtained by thresholding a real prediction $\hat{p}$, for example, $\hat{y}=\text{SGN}(\hat{p})$, can be used as base learner. The real-valued outputs ${\hat{p}}_{i}(g)$ of the calibrated classifier for node *i* on the gene *g* are viewed as estimates of the probabilities *p*_*i*_(*g*) = *ℙ*(*y*_*i*_ = 1∣*y*_par(*i*)_ = 1, *g*). The distribution of the random Boolean vector **Y** is assumed to be

(10) $$\begin{array}{c} \mathbb{P}\left(Y=y\right)=\overset{m}{\underset{i=1}{\prod }}\mathbb{P}\left(Y_{i}=y_{i}\;|\;Y_{\text{par}(i)}=1,g\right)\;\forall y\in {\left\{0,1\right\}}^{m}, \end{array}$$

where, in order to enforce that only multilabels **Y** that respect the hierarchy have nonzero probability, it is imposed that *ℙ*(*Y*_*i*_ = 1∣*Y*_par(*i*)_ = 0, *g*) = 0 for all nodes *i* = 1,…, *m* and all *g*. This implies that the base learner at node *i* is only trained on the subset of the training set including all examples (*g*, **y**) such that *y*_par(*i*)_ = 1.

#### 5.3.1. HBAYES Ensembles for Protein Function Prediction

*H*-loss is a measure of discrepancy between multilabels based on a simple intuition: if a parent class has been predicted wrongly, then errors in its descendants should not be taken into account. Given fixed cost coefficients *θ*_1_,…, *θ*_*m*_ > 0, the* H*-loss $\ell_{H}(\hat{y},v)$ between multilabels $\hat{y}$ and **v** is computed as follows: all paths in the taxonomy *T* from the root down to each leaf are examined and whenever a node *i* ∈ {1,…, *m*} is encountered such that ${\hat{y}}_{i}\neq v_{i}$, *θ*_*i*_ is added to the loss, while all the other loss contributions from the subtree rooted at *i* are discarded. This method assumes that, given a gene *g*, the distribution of the labels **V** = (*V*_1_,…, *V*_*m*_) is *ℙ*(**V** = **v**) = ∏_*i*=1_^*m*^ *p*_*i*_(*g*) for all **v** ∈ {0,1}^*m*^, where *p*_*i*_(*g*) = *ℙ*(*V*_*i*_ = *v*_*i*_∣*V*_par(*i*)_ = 1, *g*). According to the true path rule, it is imposed that *ℙ*(*V*_*i*_ = 1∣*V*_par(*i*)_ = 0, *g*) = 0 for all nodes *i* and all genes *g*.

In the evaluation phase, HBAYES predicts the Bayes-optimal multilabel $\hat{y}\in {\left\{0,1\right\}}^{m}$ for a gene *g* based on the estimates ${\hat{p}}_{i}(g)$ for *i* = 1,…, *m*. By definition of Bayes-optimality, the optimal multilabel for *g* is the one that minimizes the loss when the true multilabel **V** is drawn from the joint distribution computed from the estimated conditionals ${\hat{p}}_{i}(g)$. That is,

(11) $$\begin{array}{c} \hat{y}=\underset{y\in {\left\{0,1\right\}}^{m}}{\text{argmin}\;}𝔼\left[\ell_{H}\left(y,V\right)\;|\;g\right]. \end{array}$$

In other words, the ensemble method HBAYES provides an approximation of the optimal Bayesian classifier with respect to the* H*-loss [135]. More precisely, as shown in [27] the following theorem holds.

**Theorem 1 .**

For any tree *T* and gene *g* the multilabel generated according to the HBAYES prediction rule is the Bayes-optimal classification of *g* for the H-loss.

In the evaluation phase, the uniform cost coefficients *θ*_*i*_ = 1, for *i* = 1,…, *m*, are used. However, since with uniform coefficients the* H*-loss can be made small simply by predicting sparse multilabels (i.e., multilabels $\hat{y}$ such that $\sum_{i}{\hat{y}}_{i}$ is small), in the training phase the cost coefficients are set to *θ*_*i*_ = 1/|root(*G*)|, if *i* ∈ root(*G*), and to *θ*_*i*_ = *θ*_*j*_/|child(*j*)| with *j* = par(*i*), if otherwise. This normalizes the* H*-loss, in the sense that the maximal* H*-loss contribution of all nodes in a subtree excluding its root equals that of its root.

Let {*E*} be the indicator function of event *E*. Given *g* and the estimates ${\hat{p}}_{i}={\hat{p}}_{i}(g)$ for *i* = 1,…, *m*, the HBAYES prediction rule can be formulated as follows.

*HBAYES Prediction Rule.* Initially, set the labels of each node *i* to

(12) y^i=argminy∈{0,1}(∑j∈child(i)θip^i(1−y)+θi(1−p^i)ykkkkkkkkkk+p^i{y=1}∑j∈child(i)Hj(y^)),

where

(13) Hj(y^)=θjp^j(1−y^j)+θj(1−p^j)y^j +p^j{y^j=1}∑k∈child(j)Hk(y^)

is recursively defined over the nodes *j* in the subtree rooted at *i* with each ${\hat{y}}_{j}$ set according to (12).

Then, if ${\hat{y}}_{i}$ is set to zero, set all nodes in the subtree rooted at *i* to zero as well.

It is worth noting that $\hat{y}$ can be computed for a given *g* via a simple bottom-up message-passing procedure. It can be shown that if all child nodes *k* of *i* have ${\hat{p}}_{k}$ close to a half, then the Bayes-optimal label of *i* tends to be 0 irrespective of the value of ${\hat{p}}_{i}$. On the contrary, if *i*'s children all have ${\hat{p}}_{k}$ close to either 0 or 1, then the Bayes-optimal label of *i* is based on ${\hat{p}}_{i}$ only, ignoring the children. This behaviour can be intuitively explained in the following way: the estimate ${\hat{p}}_{k}$ is built based only on the examples on which the parent *i* of *k* is positive; hence, a “neutral” estimate ${\hat{p}}_{k}=1/2$ signals that the current instance is a negative example for the parent *i*. Experimental results show that this approach achieves comparable results with the TPR method (Section 7), an ensemble approach based on the “true path rule” [136].

#### 5.3.2. HBAYES-CS: The Cost-Sensitive Version

The HBAYES-CS is the cost-sensitive version of HBAYES proposed in [27]. By this approach, the misclassification cost coefficient *θ*_*i*_ for node *i* is split into two terms *θ*_*i*_^+^ and *θ*_*i*_^−^ for taking into account misclassifications, respectively, for positive and negative examples. By considering separately these two terms, (12) can be rewritten as

(14) y^i=argminy∈{0,1}(∑j∈child(i)θi−p^i(1−y)+θi+(1−p^i)ykkkkkkkkkk+p^i{y=1}∑j∈child(i)Hj(y^)),

where the expression of $H_{j}(\hat{y})$ gets changed correspondingly. By introducing a factor *α* ≥ 0 such that *θ*_*i*_^−^ = *αθ*_*i*_^+^ while keeping *θ*_*i*_^+^ + *θ*_*i*_^−^ = 2*θ*_*i*_, the relative costs of false positives and false negatives can be parameterized, thus allowing us to further rewrite the hierarchical Bayesian rule (Section 5.3.1) as follows:

(15) $$\begin{array}{c} {\hat{y}}_{i}=1⟺{\hat{p}}_{i}\left(2\theta_{i}-\underset{j\in \text{child}(i)}{\sum }H_{j}\right)\geq \frac{2\theta_{i}}{1+\alpha }. \end{array}$$

By setting *α* = 1, we obtain the original version of the hierarchical Bayesian ensemble and by incrementing *α* we introduce progressively lower costs for positive predictions. In this way, we can obtain that the recall of the ensemble tends to increase, eventually at the expenses of the precision, and by tuning the *α* parameter we can obtain different combinations of precision/recall values.

In principle, a cost factor *α*_*i*_ can be set for each node *i* to explicitly take into account the unbalance between the number of positive *n*_*i*_^+^ and negative *n*_*i*_^−^ examples, estimated from the training data

(16) $$\begin{array}{c} \alpha_{i}=\frac{n_{i}^{-}}{n_{i}^{+}}⟹\theta_{i}^{+}=\frac{2}{n_{i}^{-}/n_{i}^{+}+1}\theta_{i}=\frac{2n_{i}^{+}}{n_{i}^{-}+n_{i}^{+}}\theta_{i}. \end{array}$$

The decision rule (15) at each node then becomes

(17) $$\begin{array}{c} {\hat{y}}_{i}=1⟺p_{i}\left(2\theta_{i}-\underset{j\in \text{child}\left(i\right)}{\sum }H_{j}\right)\geq \frac{2\theta_{i}}{1+\alpha_{i}}=\frac{2\theta_{i}n_{i}^{+}}{n_{i}^{-}+n_{i}^{+}}. \end{array}$$

Results obtained with the yeast model organism showed that HBAYES-CS significantly outperform HTD methods [27, 136].

## 6. Reconciliation Methods

Hierarchical ensemble methods are basically two-step methods, since at first provide predictions for the single classes and then arrange these predictions to take into account the functional relationships between GO terms. Noble and colleagues name this general approach* reconciliation methods* [24]: they proposed methods for calibrating and combining independent predictions to obtain a set of probabilistic predictions that are consistent with the topology of the ontology. They applied their ensemble methods to the genome-wide and ontology-wide function prediction with* M. musculus*, involving about 3000 GO terms.

Their goal consists in providing consistent predictions, that is, predictions whose confidence (e.g., posterior probability) increases as we ascend from more specific to more general terms in the GO. Moreover, another important issue of these methods is the availability of confidence values associated with the predictions that can be interpreted as probabilities that a protein has a certain function given the information provided by the data.

**Figure 10:**
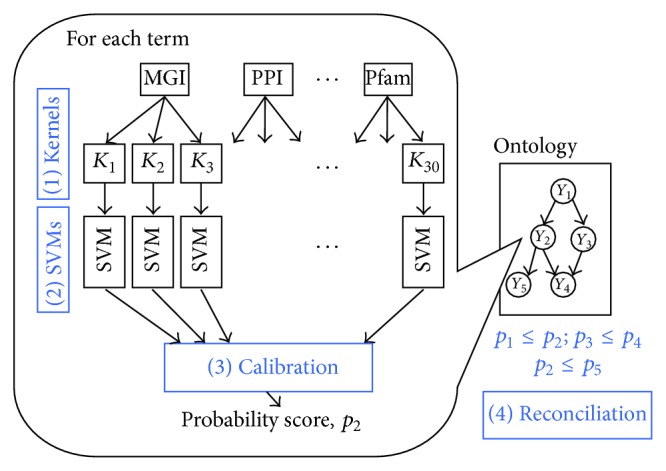
The overall scheme of reconciliation methods (adapted from [24]).

The overall reconciliation approach can be summarized in the following four basic steps (Figure 10):

1. *Kernel computation*: at first a set of kernels is computed from the available data. We may choose kernel specific for each source of data (e.g., diffusion kernels for protein-protein interaction data [137], linear or Gaussian kernel for expression data, and string kernel for sequence data [138]). Multiple kernels for the same type of data can also be constructed [24].
2. *SVM learning*: SVMs are used as base learners using the kernels selected at the previous step; the training is performed by internal cross-validation to avoid overfitting, and a local cost-sensitive strategy is applied, by tuning separately the *C* regularization factor for positive and negative examples. Note that the authors in their experiments used SVMs as base learners but any meaningful classifier could be used at this step.
3. *Calibration*: to produce individual probabilistic outputs from the set of SVM outputs corresponding to one GO term, a logistic regression approach is applied. In this way, a calibration of the individual SVM outputs is obtained, resulting in a probabilistic prediction of the random variable *Y*_*i*_, for each node/term *i* of the hierarchy, given the outputs ${\hat{y}}_{i}$ of the SVM classifiers.
4. *Reconciliation*: the first three steps generate unreconciled outputs; that is, in practice a “flat” ensemble is applied that may generate inconsistent predictions with respect to the given taxonomy. In this step, the outputs of step three are processed by a “reconciliation method.” The goal of this stage is to combine predictions for each term to produce predictions that are consistent with the ontology, meaning that all the probabilities assigned to the ancestors of a GO term are larger than the probability assigned to that term.

The first three steps are basically the same for (or very similar to) each reconciliation ensemble method. The crucial step is represented by the fourth, that is, the reconciliation step, and different ensemble algorithms can be designed to implement it. The authors proposed 11 different ensemble methods for the reconciliation of the base classifier outputs. Schematically, they can be subdivided into the following four main classes of ensembles:

1. heuristic methods;
2. Bayesian network-based methods;
3. cascaded logistic regression;
4. projection-based methods.

### 6.1. Heuristic Methods

These approaches preserve the “reconciliation property”

(18) $$\begin{array}{c} \forall i,j\in G,\;\;\left(i,j\right)\in G⟹{\hat{p}}_{i}\geq {\hat{p}}_{j} \end{array}$$

through simple heuristic modifications of the probabilities computed at step 3 of the overall reconciliation scheme.

- The MAX method simply chooses the largest logistic regression value for the node *i* and all its descendants desc
  (19) $$\begin{array}{c} p_{i}=\underset{j\in \text{desc}(i)}{\max}{\hat{p}}_{j}. \end{array}$$
- The AND method implements the notion that the probability of all ancestral GO terms anc(*i*) of a given term/node *i* is large, assuming that, conditional on the data, all predictions are independent
  (20) $$\begin{array}{c} p_{i}=\underset{j\in \text{anc}(i)}{\prod }{\hat{p}}_{j}. \end{array}$$
- OR estimates the probability that the node *i* is annotated at least for one of the descendant GO terms, assuming again that, conditional on the data, all predictions are independent
  (21) $$\begin{array}{c} 1-p_{i}=\underset{j\in \text{desc}\left(i\right)}{\prod }\left(1-{\hat{p}}_{j}\right). \end{array}$$

### 6.2. Cascaded Logistic Regression

Instead of modeling class-conditional probabilities, as required by the Bayesian approach, logistic regression can be used instead to directly model posterior probabilities. Considering that modeling conditional densities are in most cases difficult (also using strong independence assumptions as shown in Section 5.1), the choice of logistic regression could be a reasonable one. In [24], the authors embedded in the logistic regression setting the hierarchical dependencies between terms. By assuming that a random variable **X** whose values represent the features of the gene *g* of interest is associated to a given gene *g* and assuming that *ℙ*(**Y** = **y**∣**X** = **x**) factorizes according to the GO graph, then it follows

(22) ℙ(Y=y ∣ X=x) =∏iℙ(Yi=yi ∣ ∀j∈par(i)Yj=yj,Xi=xi)

with *ℙ*(*Y*_*i*_ = 1∣∀ *j* ∈ par(*i*)  *Y*_*j*_ = 0, *X*_*i*_ = *x*_*i*_) = 0. The authors estimated *ℙ*(*Y*_*i*_ = 1∣∀ *j* ∈ par(*i*)*Y*_*j*_ = 1, *X*_*i*_ = *x*_*i*_) with logistic regression. This approach is quite similar to fitting independent logistic regressions, but note that in this case only examples of proteins having all parents GO terms are used to fit the model, thus implicitly taking into account the hierarchical relationships between GO terms.

### 6.3. Bayesian Network-Based Methods

These methods are variants of the Bayesian network approach proposed in [20] (Section 5.1): the GO is viewed as a graphical model where a joint Bayesian prior is put on the binary GO term variables *y*_*i*_. The authors proposed four variants that can be summarized as follows:

- the BPAL is a belief propagation approach with asymmetric Laplace likelihoods. The graphical model has edges directed from more general terms to more specific terms. Differently from [20], the distribution of each SVM output is modeled as an asymmetric Laplace distribution, and a variational inference algorithm that solves an optimization problem whose minimizer is the set of marginal probabilities of the distribution is used to estimate the posterior probabilities of the ensemble [139];
- the BPALF approach is similar to BPAL but with edges inverted and directed from more specific terms to more general terms;
- the BPLR is a heuristic variant of BPAL, where, in the inference algorithm, the Bayesian log posterior ratio for *Y*_*i*_ is replaced by the marginal log posterior ratio obtained from the logistic regression (LR);
- The BPLRF is equal to BPLR but with reversed edges.

### 6.4. Projection-Based Methods

A different approach is represented by methods that directly use the calibrated values obtained from logistic regression (step 3 of the overall scheme of the reconciliation methods) to find the closest set of values that are consistent with the ontology. This approach leads to a constrained optimization problem. The main contribution of the Obozinski et al. work [24] is represented by the introduction of projection reconciliation techniques based on isotonic regression [140] and the Kullback-Leibler divergence.

The* isotonic regression* method tries to find a set of marginal probabilities *p*_*i*_ that are close to the set of calibrated values ${\hat{p}}_{i}$ obtained from the logistic regression. The Euclidean distance is used as a measure of closeness. Hence, considering that the “reconciliation property” requires that *p*_*i*_ ≥ *p*_*j*_ when (*i*, *j*) ∈ *E*, this approach yields the following quadratic program:

(23) min⁡pi,i∈I ∑i∈I(pi−p^i)2s.t.kkkpj≤pi, (i,j)∈E.

This problem is the classical isotonic regression problems that can be solved using an interior point solver or also approximated algorithm when the number of edges of the graph is too large [141].

Considering that we deal with probabilities, a natural measure of distance between probability density functions *f*(**x**) and *g*(**x**) defined with respect to a random variable **x** is represented by the Kullback-Leibler divergence *D*_*f*_**x**_||*g*_**x**__ as follows:

(24) $$\begin{array}{c} D_{f_{x}||g_{x}}=\overset{\infty }{\underset{-\infty }{\int }}f\left(x\right)\log\left(\frac{f\left(x\right)}{g\left(x\right)}\right)dx. \end{array}$$

In the context of reconciliation methods, we need to consider a discrete version of the Kullback-Leibler divergence, yielding the following optimization problem:

(25) min⁡p ⁡Dp^||p=min⁡pi,i∈I⁡∑i∈Ip^ilog⁡(p^ipi)s.t.kkpj≤pi, (i,j)∈E.

The algorithm finds the probabilities closest to the probabilities $\hat{p}$ obtained from logistic regression according to the Kullback-Leibler divergence and obeying the constraints that probabilities cannot increase while descending on the hierarchy underlying the ontology.

The extensive experiments exploited in [24] show that, among the reconciliation methods, isotonic regression is the most generally useful. Across a range of evaluation modes, term sizes, ontologies, and recall levels, isotonic regression yields consistently high precision. On the other hand, isotonic regression is not always the “best method,” and a biologist with a particular goal in mind may apply other reconciliation methods. For instance, with small terms usually Kullback-Leibler projections achieve the best results, but considering average “per term” results heuristic methods yield precision at a given recall comparable with projection methods and better than that achieved with Bayes-net methods.

This ensemble approach achieved excellent results in the prediction of protein function in the mouse model organism, demonstrating that hierarchical multilabel methods can play a crucial role for the improvement of protein function prediction performances [24]. Nevertheless, the approach suffers from some drawbacks. Indeed, the paper focuses on the comparison of hierarchical multilabel methods, but it does not analyze impact of the concurrent use of data integration and hierarchical multilabel methods on the overall classification performances. Moreover, potential improvements could be introduced by applying cost-sensitive variants of hierarchical multilabel predictors, able to effectively calibrate the precision/recall trade-off at different levels of the functional ontology.

## 7. True Path Rule Hierarchical Ensembles

These ensemble methods exploit at the same time the downward and upward relationships between classes, thus considering both the parent-to-child and child-to-parent functional links (Figure 2(b)).

The true path rule (TPR) ensemble method [31, 142] is directly inspired by the* true path rule* that governs both GO and FunCat taxonomies. Citing the curators of the Gene Ontology is as follows [143]: “An annotation for a class in the hierarchy is automatically transferred to its ancestors, while genes unannotated for a class cannot be annotated for its descendants.” Considering the parents of a given node *i*, a classifier that respects the true path rule needs to obey the following rules:

(26) $$\begin{array}{c} y_{i}=1⟹y_{\text{par}(i)}=1\\ y_{i}=0⇏y_{\text{par}(i)}=0. \end{array}$$

On the other hand, considering the children of a given node *i*, a classifier that respects the true path rule needs to obey the following rules:

(27) $$\begin{array}{c} y_{i}=1⇏y_{\text{child}(i)}=1\\ y_{i}=0⟹y_{\text{child}(i)}=0. \end{array}$$

From (26) and (27), we observe an asymmetry in the rules that govern the assignments of positive and negative labels. Indeed, we have a propagation of positive predictions from bottom to top of the hierarchy in (26) and a propagation of negative labels from top to bottom in (27). Conversely, negative labels cannot propagate from bottom to top, and positive predictions cannot propagate from top to bottom.

**Figure 11:**
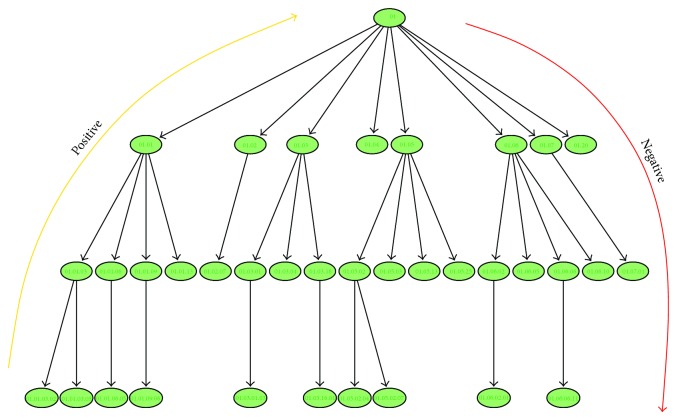
The asymmetric flow of information suggested by the true path rule.

The “true path rule” suggests algorithms able to propagate “positive” decisions from bottom to top of the hierarchy and negative decisions from top to bottom (Figure 11).

### 7.1. The True Path Rule Ensemble Algorithm

The TPR algorithm puts together the predictions made at each node by local “base” classifiers to realize an ensemble that obeys the “true path rule.”

The basic ideas behind the method can be summarized as follows:

1. training of the base learners: for each node of the hierarchy, a suitable learning algorithm (e.g., a multilayer perceptron or a support vector machine) provides a classifier for the associated functional class;
2. in the evaluation phase, the trained classifiers associated with each class/node of the graph provide a local decision about the assignment of a given example to a given node;
3. positive decisions, that is, annotations to a specific functional class, may propagate from bottom to top across the graph: they influence the decisions of the parent nodes and of their ancestors in a recursive way, by traversing the graph towards higher level nodes/classes. Conversely, negative decisions do not affect decisions of the parent node;that is, they do not propagate from bottom to top (26);
4. negative predictions for a given node (taking into account the local decision of its descendants) are propagated to the descendants, to preserve the consistency of the hierarchy according to the true path rule, while positive decisions do not influence decisions of child nodes (27).

The ensemble combines the local predictions of the base learners associated with each node with the positive decisions that come from the bottom of the hierarchy, and with the negative decisions that spring from the higher level nodes. More precisely, base classifiers estimate local probabilities ${\hat{p}}_{i}(g)$ that a given example *g* belongs to class *θ*_*i*_, but the core of the algorithm is represented by the evaluation phase, where the ensemble provides an estimate of the “consensus” global probability ${\bar{p}}_{i}(g)$.

It is worth noting that instead of a probability, ${\hat{p}}_{i}(g)$ may represent a score associated with the likelihood that a given gene/gene product belongs to the functional class *i*.

Let us consider the set *ϕ*_*i*_(*g*) of the children of node *i* for which we have a positive prediction for a given gene *g*

(28) $$\begin{array}{c} \phi_{i}\left(g\right)=\left\{j:j\in \text{child}\left(i\right),{\hat{y}}_{j}=1\right\}. \end{array}$$

The global consensus probability ${\bar{p}}_{i}(g)$ of the ensemble depends both on the local prediction ${\hat{p}}_{i}(g)$ and on the prediction of the nodes belonging to *ϕ*_*i*_(*g*)

(29) $$\begin{array}{c} {\bar{p}}_{i}\left(g\right)=\frac{1}{1+\left|\phi_{i}\left(g\right)\right|}\left({\hat{p}}_{i}\left(g\right)+\underset{j\in \phi_{i}(g)}{\sum }{\bar{p}}_{j}\left(g\right)\right). \end{array}$$

The decision ${\hat{y}}_{i}(g)$ at node/class *i* is set to 1 if ${\bar{p}}_{i}(g)>t$ and to 0, if otherwise (a natural choice for *t* is 0.5), and only children nodes for which we have a positive prediction can influence their parent. In the leaf nodes, the sum of (29) disappears and (29) becomes ${\bar{p}}_{i}(g)={\hat{p}}_{i}(g)$. In this way, positive predictions propagate from bottom to top, and negative decisions are propagated to their descendants when for a given node ${\hat{y}}_{i}(g)=0$.

The bottom-up per level traversal of the tree assures that all the offsprings of a given node *i* are taken into account for the ensemble prediction. For the same reason, we can safely set the classes belonging to the subtree rooted at *i* to negative, when ${\hat{y}}_{i}$ is set to 0. It is worth noting that we have a two-way asymmetric flow of information across the tree: positive predictions for a node influence its ancestors, while negative predictions influence its offsprings.

The algorithm provides both the multilabels ${\hat{y}}_{i}$ and an estimate of the probabilities ${\bar{p}}_{i}$ that a given example *g* belongs to the class *i* = 1,…, *m*.

### 7.2. The Cost-Sensitive Variant

Note that in the TPR algorithm there is no way to explicitly balance the local prediction ${\hat{p}}_{i}(g)$ at node *i* with the positive predictions coming from its offsprings (29). By balancing the local predictions with the positive predictions coming from the ensemble, we can explicitly modulate the interplay between local and descendant predictors. To this end, a* weight w*, 0 ≤ *w* ≤ 1, is introduced, such that if *w* = 1 the decision at node *i* depends only by the local predictor; otherwise, the prediction is shared proportionally to *w* and 1 − *w* between, respectively, the local parent predictor and the set of its children

(30) $$\begin{array}{c} {\bar{p}}_{i}=w{\hat{p}}_{i}+\frac{1-w}{\left|\phi_{i}\right|}\underset{j\in \phi_{i}}{\sum }{\bar{p}}_{j}. \end{array}$$

This variant of the TPR algorithm is the* weighted true path rule *(TPR-W) hierarchical ensemble algorithm. By tuning the *w* parameter, we can modulate the precision/recall characteristics of the resulting ensemble. More precisely, for *w* → 0, the weight of the parent local predictor is small, and the ensemble decision mainly depends on the positive predictions of the offsprings nodes (classifiers). Conversely, *w* → 1 corresponds to a higher weight of the parent predictor; then, less weight is given to possible positive predictions of the children, and the decision depends mainly on the local/parent base classifier. In case of a negative decision, all the subtree is set to 0, causing the precision to increase. Note that for *w* → 1 the behaviour of TPR-W becomes similar to that of HTD (Section 4).

A specific advantage of the TPR-W ensembles is the capability of tuning precision and recall rates, through the parameter *w* (30). For small values of *w*, the weight of the decision of the parent local predictor is small, and the ensemble decision depends mainly by the positive predictions of the offsprings nodes (classifiers), and higher values of *w* correspond to a higher weight of the “parent” local predictor, with a resulting higher precision. In [31], the author shows that the *w* parameter highly influences the precision/recall characteristics of the ensemble: low *w* values yield a higher recall, while high values improve the precision of the TPR-W ensemble.

Recently, Chen and Hu proposed a method that applies the TPR-W hierarchical strategy, but using composite kernel SVMs as base classifiers, and a supervised clustering with oversampling strategy to solve the imbalance data set learning problem, showed that the proper selection of base learners, and unbalance-aware learning strategies can further improve the results in terms of hierarchical precision and recall [144].

The same authors proposed also an enhanced version of the TPR-W strategy to overcome a limitation of this bottom-up hierarchical method for AFP. Indeed, for some classes at the lower levels of the hierarchy, the classifier performances are sometimes quite poor, due to both noisy data and the relatively low number of available annotations. More precisely, in the basic TPR ensemble, the probabilities ${\bar{p}}_{j}$ computed by the children of the node *i* (30) contribute in equal way to the probability ${\bar{p}}_{i}$ computed by the ensemble at node *i*, independently of the accuracy of the predictions made by its children classifiers. This “unweighted” mechanism may generate error propagation of the errors across the hierarchy: a poor performance child classifier may, for instance, with high probability, predict a negative example as positive and this error may propagate to its parent node and recursively to its ancestor nodes. To try to alleviate this possible bottom-up error propagation in [145], Chen and Hu proposed an improved TPR ensemble (TPR-W* weighted*), based on classifier performance. To this end, they weighted the contribution of each child classifier on the basis of their performance evaluated on a validation data set, by adding to (30) another weight *ν*_*j*_

(31) $$\begin{array}{c} {\bar{p}}_{i}=w{\hat{p}}_{i}+\frac{1-w}{\left|\phi_{i}\right|}\underset{j\in \phi_{i}}{\sum }\nu_{j}\cdot {\bar{p}}_{j}, \end{array}$$

where *ν*_*j*_ is computed on the basis of some accuracy metric *A*_*j*_ (e.g., the* F*-score) estimated for the child classifiers associated with node *j* as follows:

(32) $$\begin{array}{c} \nu_{j}=\frac{A_{j}}{\sum_{k\in \phi_{i}}A_{k}}. \end{array}$$

In this way, the contribution of “poor” classifier is reduced, while “good” classifiers weight more in the final computation of ${\bar{p}}_{i}$ (31). Experiments with the “Protein Fate” subtree of the FunCat taxonomy with the yeast model organism show that this approach improves prediction with respect to the “vanilla” TPR-W hierarchical strategy [145].

### 7.3. Advantages and Drawbacks of TPR Methods

While the propagation of negative decisions from top to bottom nodes is quite straightforward and common to the hierarchical* top-down* algorithm, the propagation of positive decisions from bottom to top nodes of the hierarchy is specific to the TPR algorithm. For a discussion of this item, see Appendix C.

Experimental results show that TPR-W achieves equal or better results than the TPR and* top-down* hierarchical strategy, and both hierarchical strategies achieve significantly better results than* flat* classification methods [55, 123]. The analysis of the per level classification performances shows that TPR-W, by exploiting a global strategy of classification, is able to achieve a good compromise between precision and recall, enhancing the* F*-measure at each level of the taxonomy [31].

Another advantage of TPR-W consists in the possibility of tuning precision and recall by using a global strategy: large values of the *w* parameter improve the precision, and small values improve the recall.

Moreover, TPR and TPR-W ensembles provide also a probabilistic estimate of the prediction reliability for each functional class of the overall taxonomy.

The decisions performed at each node of the hierarchical ensemble are influenced by the positive decisions of its descendants. More precisely, the analyses performed in [31] showed the following:

- weights of descendants decrease exponentially with respect to their depth. As a consequence the influence of descendant, nodes decay quickly with their depth;
- the parameter *w* plays a central role in balancing the weight of the parent classifier associated with a given node with the weights of its positive offsprings: small values of *w* increase the weight of descendant nodes, and large values increase the weight of the local parent predictor associated with that node;
- the effect on the overall probability predicted by the ensemble is the result of the choice of the *w* parameter, the strength of the prediction of the local learners and of its descendants.

These characteristics of TPR-W ensembles are well suited for the hierarchical classification of protein functions, considering that annotations of deeper nodes are likely to have less experimental evidence than higher nodes. Moreover, by enforcing the strength of the descendant nodes through low *w* values, we can improve the recall characteristics of the overall system (at the expense of a possible reduction in precision).

Unfortunately, the method has been conceived and applied only to the FunCat taxonomy, structured according to a tree forest (Section 11), while no applications have been performed using the GO, structured according to a directed acyclic graph (Section 11).

## 8. Ensembles Based on Decision Trees

Another interesting research line is represented by hierarchical methods base on inductive decision trees [146]. The first attempts to exploit the hierarchical structure of functional ontologies for AFP simply used different decision tree models for each level of the hierarchy [147] or investigated a modified decision tree model, in which the assignment to a node is propagated toward the parent nodes [9], by extending the classical C4.5 decision tree algorithm for multiclass classification.

In the context of the predictive clustering tree framework [148], Blockeel et al. proposed an improved version which they applied to the prediction of gene function in the yeast [149].

More recent approaches, always based on modified decision trees, used distance measure derived from the hierarchy and significantly improved previous methods [54]. The authors showed that separate decision tree models are less accurate than a single decision tree trained to predict all classes at once, even when they are built taking into account the hierarchy.

Nevertheless, the previously proposed decision tree-base methods often achieve results not comparable with state-of-the-art hierarchical ensemble methods. To overcome this limitation, Schietgat et al. showed that ensembles of hierarchical multilabel decision trees are competitive with state-of-the-art statistical learning methods for DAG-structured prediction of protein function in* S. cerevisiae*,* A. thaliana,* and* M. musculus* model organisms [29]. A further work explored the suitability of different ensemble methods based on predictive clustering trees, ranging from global ensembles that learn ensembles of predictive models, each able to predict the entire structure of the hierarchy (i.e., all the GO terms for a given gene), to local ensembles that train an entire ensemble as a classifier for each branch of the taxonomy. Recently, a novel approach used PPI network autocorrelation in hierarchical multilabel classification trees to improve gene function prediction [150].

In [151], methods related to decision trees, in the sense that interpretable classification rules to predict all functions at all levels of the GO hierarchy, have been proposed, using an ant colony optimization classification algorithm to discover classification rules.

Finally, bagging and random forest ensembles [152] have been applied to the AFP in yeast, showing that both local and global hierarchical ensemble approaches perform better than the single model counterparts in terms of predictive power [153].

## 9. The Hierarchical Classification Alone Is Not Enough

Several works showed that in protein function prediction problems we need to consider several learning issues [1, 16, 18]. In particular, in [80], the authors showed that even if hierarchical ensemble methods are fundamental to improve the accuracy of the predictions, their mere application is not enough to assure state-of-the-art results if we at the same time do not consider other important learning issues related to AFP. Indeed, in [123], it has been shown a significant synergy between hierarchical classification, data integration methods, and cost-sensitive techniques, highlighting that hierarchical ensemble methods should be designed taking into account different learning issues essential for the AFP problem.

### 9.1. Hierarchical Methods and Data Integration

Several works and the recently published results of the CAFA 2011 (Critical Assessment of Functional Annotation) challenge showed that data integration plays a central role to improve the predictions of protein functions [16, 25, 154–156].

Indeed, high-throughput biotechnologies make increasing quantities of biomolecular data of different types available, and several works pointed out that data integration is fundamental to improve the accuracy in AFP [1].

According to [154], we may subdivide the main approaches to data integration for AFP in four groups as follows:

1. vector subspace integration;
2. functional association networks integration;
3. kernel fusion;
4. ensemble methods.

*Vector Space Integration*. This approach consists in concatenating vectorial data to combine different sources of biomolecular data [132]. For instance, [22] concatenates different vectors, each one corresponding to a different source of genomic data, in order to obtain a larger vector that is used to train a standard SVM. A similar approach has been proposed by [30], but each data source is separately normalized in order to take into account the data distribution in each individual vector space.

*Functional Association Networks Integration*. In functional association networks, different graphs are combined to obtain the composite resulting network [21, 48]. The simplest approaches adopt conjunctive/disjunctive techniques [63], that is, respectively, adding an edge when in all the networks two genes are linked together or when a link between the two genes is present in at least one functional network or probabilistic evidence integration schemes [45].

Other methods differentially weight each data source using techniques ranging from Gaussian random fields [46] to the naive-Bayes integration [157] and constrained linear regression [14], or by merging data taking into account the GO hierarchy [158], or by applying XML-based techniques [159].

*Kernel Fusion.* These techniques at first construct a separated Gram matrix for each available data source using appropriate kernels representing similarities between genes/gene products. Then, by exploiting the closure property with respect to the sum and other algebraic operators, the Gram matrices are combined to obtain a “consensus” global integrated matrix.

Besides combining kernels linearly with fixed coefficients [22], one may also use semidefinite programming to learn the coefficients [11]. As methods based on semidefinite programming do not scale well to multiple data sources, more efficient methods for multiple kernel learning have been recently proposed [160, 161]. Kernel fusion methods, both with and without weighting the data sources, have been successfully applied to the classification of protein functions [162–165]. Recently, a novel method proposed an enhanced kernel integration approach by which the weights are iteratively optimized by reducing the empirical loss of a multilabel classifier for each of the labels simultaneously, using a combined objective function [165].

*Ensemble Methods.* Genomic data fusion can be realized by means of an ensemble system composed by learners trained on different “views” of the data and then combining the outputs of the component learners. Each type of data may capture different and complementary characteristics of the objects to be classified and the resulting ensemble may obtain better prediction capabilities through the diversity and the anticorrelation of the base learner responses.

Some examples of ensemble methods for data combination include “late integration” of kernels trained on different sources [22], the naive-Bayes integration [166] of the outputs of SVMs trained with multiple sources [30], and logistic regression for combining the output of several SVMs trained with different biomolecular data and kernels [24].

Recently, in [23], the authors showed that simple ensemble methods, such as weighted voting [167, 168] or decision templates [169], give results comparable to state-of-the-art data integration methods, exploiting at the same time the modularity and scalability that characterize most ensemble algorithms. Another work showed that ensemble methods are also resistant to noise [170].

Using an ensemble approach, biomolecular data differing in their structural characteristics (e.g., sequences, vectors, and graphs) can be easily integrated, because with ensemble methods the integration is performed at the decision level, combining the outputs produced by classifiers trained on different datasets [171–173].

As an example of the effectiveness of the integration of hierarchical ensemble methods with data fusion techniques, in [123], six different sources of yeast biomolecular data have been integrated, ranging from protein domain data (PFAM BINARY and PFAM LOGE) [174], gene expression measures (EXPR) [175], predicted and experimentally supported protein-protein interaction data (STRING and BIOGRID) [176, 177] to pairwise sequence similarity data (SEQ. SIM.). Kernel fusion integration (sum of the Gram matrices) has been applied, and preprocessing has been performed using the the* HCGene* R package [178].

Table 2 summarizes the results of the comparison across about two hundreds of FunCat classes, including single-source and data integration approaches together with both flat and hierarchical ensembles.

Data fusion techniques improve average per class* F*-score across classes in flat ensembles (first column of Table 2) and significantly boost multilabel hierarchical methods (columns HTD, HTD-CS, HB, HB-CS, TPR, and TPR-W of Table 2).

**Figure 12:**
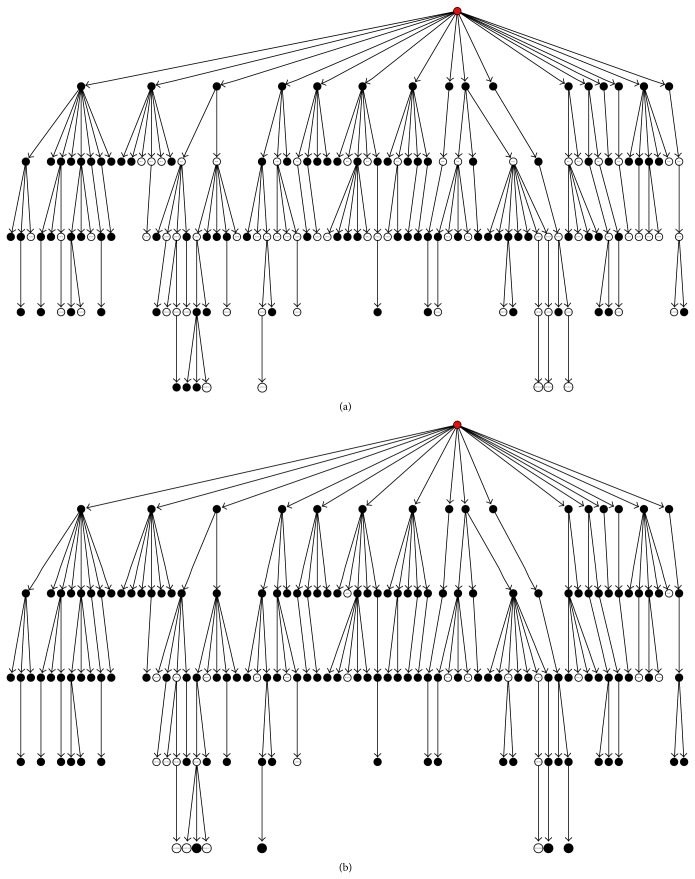
FunCat trees to compare* F*-scores achieved with data integration (KF) to the best single-source classifiers trained on BIOGRID data. Black nodes depict functional classes for which KF achieves better* F*-scores. (a) FLAT and (b) TPR-W ensembles.

Figure 12 depicts the classes (black nodes) where kernel fusion achieves better results than the best single-source data set (BIOGRID). It is worth noting that the number of black nodes is significantly larger in TPR-W (Figure 12(b)) with respect to FLAT methods (Figure 12(a)).

Hierarchical multilabel ensembles largely outperform FLAT approaches [24, 30], but Table 2 and Figure 12 also reveal a synergy between hierarchical ensemble methods and data fusion techniques.

### 9.2. Hierarchical Methods and Cost-Sensitive Techniques

**Figure 13:**
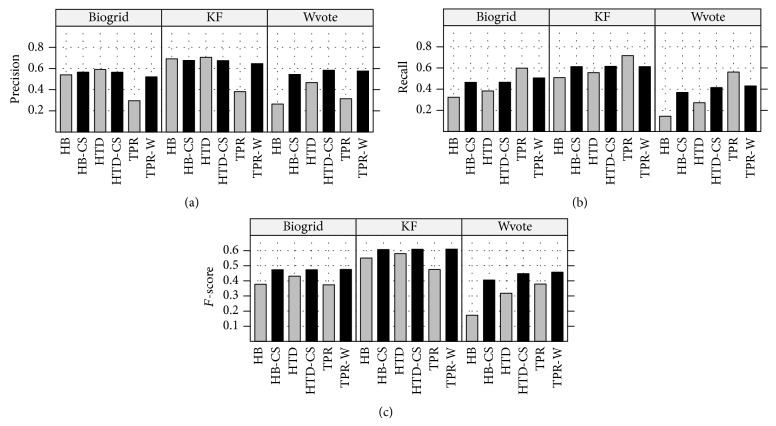
Comparison of hierarchical precision, recall, and* F*-score among different hierarchical ensemble methods using the best source of biomolecular data (BIOGRID), kernel fusion (KF), and weighted voting (WVOTE) data integration techniques. HB stands for HBAYES.

According to [27, 142], cost-sensitive approaches boost predictions of hierarchical methods when single-sources of data are used to train the base learners. These results are confirmed when cost-sensitive methods (HBAYES-CS, Section 5.3.2; HTD-CS, Section 4; and TPR-W, Section 7.2) are integrated with data fusion techniques, showing a synergy between multilabel hierarchical, data fusion (in particular, kernel fusion), and cost-sensitive approaches (Figure 13) [123].

**Figure 14:**
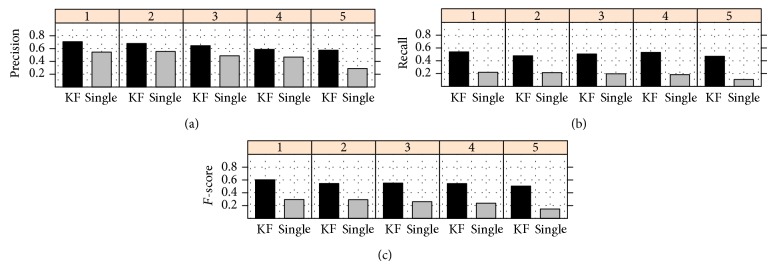
Comparison of per level average precision, recall, and* F*-score across the five levels of the FunCat taxonomy in HBAYES-CS using single data sets (single) and kernel fusion techniques (KF). Performance of “single” is computed by averaging across all the single data sources.

Perlevel analysis of the* F*-score in HBAYES-CS, HTD-CS, and TPR-W ensembles shows a certain degradation of performance with respect to the depth of nodes, but this degradation is significantly lower when data fusion is applied. Indeed, the per-level* F*-score achieved by HBAYES-CS and HTD-CS when a single source is used consistently decreases from the top to the bottom level, and it is halved at level 5 with respect to the first level. On the other hand, in the experiments with Kernel Fusion the average* F*-score at level 2, 3 and 4 is comparable, and the decrement at level 5 with respect to level 1 is only about 15% (Figure 14). Similar results are reported also with TPR-W ensembles.

In conclusion, the synergic effects of hierarchical multilabel ensembles, cost-sensitive, and data fusion techniques significantly improve the performance of AFP. Moreover, these enhancements allow obtaining better and more homogeneous results at each level of the hierarchy. This is of paramount importance, because more specific annotations are more informative and can get more biological insights into the functions of genes.

### 9.3. Different Strategies to Select “Negative” Genes

In both GO and FunCat, only positive annotations are usually available, while negative annotations are much reduced. More precisely, in the GO, only about 2500 negative annotations are available, and surely this amount does not allow a sufficient coverage of negative examples.

Moreover, some seminal works in functional genomics pointed out that the strategy of choosing negative training examples does affect the classifier performance [8, 163, 179, 180].

In [123], two strategies for choosing negative examples have been compared: the* basic* (*B*) and the* parent only* (PO) strategy.

According to the *B* strategy, the set of negative examples are simply those genes *g* that are not annotated for class *c*_*i*_; that is,

(33) $$\begin{array}{c} N_{B}=\left\{g:g\notin c_{i}\right\}. \end{array}$$

The PO selection strategy chooses as negatives for the class *c*_*i*_ only those examples that are nonannotated to *c*_*i*_ but are annotated for a parent class. More precisely, for a given class *c*_*i*_ corresponding to node *i* in the taxonomy, the set of negative examples is

(34) $$\begin{array}{c} N_{\text{PO}}=\left\{g:g\notin c_{i},g\in \text{par}\left(i\right)\right\}. \end{array}$$

Hence, this strategy selects negative examples for training that are in a certain sense “close” to positives. It is easy to see that *N*_PO_⊆*N*_*B*_; hence, this strategy selects for training a large set of generic negative examples, possibly annotated with classes that are associated with faraway nodes in the taxonomy. Of course, the set of positive examples is the same for both strategies.

**Figure 15:**
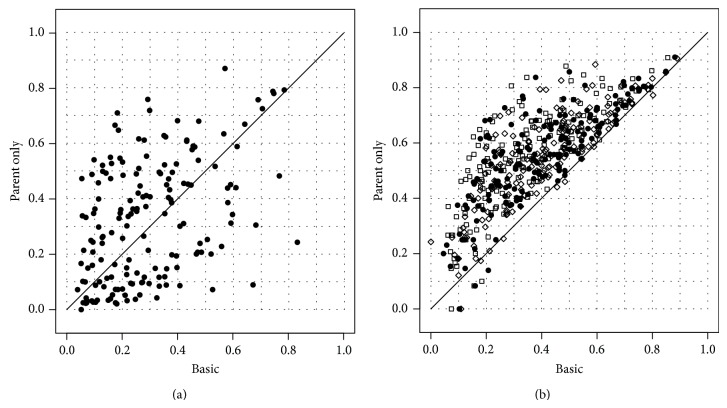
Comparison of average per class* F*-score between* basic* and PO strategies. (a) FLAT ensembles; (b) hierarchical cost-sensitive strategies: HTD-CS (squares), TPR-W (triangles), and HBAYES-CS (filled circles). Abscissa: per class* F*-score with base learners trained according to the* basic* strategy; ordinate: per class* F*-score with base learners trained according to the PO strategy.

The *B* strategy worsens the performance of hierarchical multilabel methods, while for FLAT ensembles there is no clear trend. Indeed, in Figure 15, we compare the* F*-scores obtained with *B* to those obtained with PO, using both hierarchical cost-sensitive (Figure 15(a)) and FLAT (Figure 15(b)) methods. Each point represents the* F*-score for a specific FunCat class achieved by a specific method with* B* (abscissa) and PO (ordinate) strategy for the selection of negative examples. In Figure 15(a), most points lie above the bisector independently of the hierarchical cost-sensitive method being used. This shows that hierarchical methods gain in performance when using the PO strategy as opposed to the *B* strategy (*P*-value = 2.2 × 10^−16^ according to the Wilcoxon signed-ranks test). This is not the case for FLAT methods (Figure 15(b)).

These results can be explained by considering that the PO strategy takes into account the hierarchy to select negatives, while the *B* strategy does not. More precisely, FLAT methods having no information about the hierarchical structure of classes may fail to distinguish negative examples belonging to very distant classes, thus resulting in a high false positive rate, while hierarchical methods, which know the taxonomy, can use the information coming from other base classifiers to prevent a local base learner from incorrectly classifying “distant” negative examples.

In conclusion, these seminal works show that the strategy to choose negative examples exerts a significant impact on the accuracy of the predictions of hierarchical ensemble methods, and more research work is needed to explore this topic.

## 10. Open Problems and Future Trends

In the previous section, we showed that different learning issues should be considered to improve the effectiveness and the reliability of hierarchical ensemble methods. Most of these issues and others related to hierarchical ensemble methods and to AFP represent challenging problems that have been only partially considered by previous work. For these reasons, we try to delineate some of the open problem and research trends in the context of this research area.

For instance, the selection strategies for negative examples have been only partially explored, even if some seminal works show that this item exerts a significant impact on the accuracy of the predictions [123, 163, 179, 180]. Theoretical and experimental comparison of different strategies should be performed in a systematic way, to assess the impact of the different strategies on different hierarchical methods, considering also the characteristics of the learning machines used as base learners.

Some works showed also that the cost-sensitive strategies are needed to significantly improve predictions, especially in a hierarchical context [123], but new research could be considered for both applying and designing cost-sensitive base learners and to develop novel hierarchical ensemble unbalance-aware. Cost-sensitive methods have been applied to both the single base learners and also to the overall hierarchical ensemble strategy [31, 123], and recently a hierarchical variant of* SMOTE* (synthetic minority oversampling technique) [181] has been applied to hierarchical protein function prediction, showing very promising results [145]. In principle classical “balancing” strategies should be explored to improve the accuracy and the reliability of the base learners and hence of the overall hierarchical classification process. For instance, random undersampling or oversampling techniques could be applied: the former augments the annotations by exactly duplicating the annotated proteins, whereas the latter randomly takes away some unannotated examples [182]. Other approaches could be considered such as heuristic resampling methods [183] or embedding resampling methods into data mining algorithms [184] or ensemble methods tailored to imbalanced classification problems [185–187].

Since functional classes are unbalanced, precision/recall analysis plays a central role in AFP problems and often drives “in vitro” experiments that provide biological insights into specific functional genomics problems [1]. Only a few hierarchical ensemble methods, such as HBAYES-CS [27] and TPR-W [31], can tune their precision/recall characteristics through a single global parameter. In HBAYES-CS, by incrementing the cost factor *α* = *θ*_*i*_^−^/*θ*_*i*_^+^, we introduce progressively lower costs for positive predictions, thus resulting in an increment of the recall (at the expenses of a possibly lower precision). In TPR-W, by incrementing *w*, we can reduce the recall and enhance the precision. Parametric versions of other hierarchical ensemble methods could be developed, in order to design ensemble methods with “tunable” precision/recall characteristics.

Another important issue that should be considered in the design of novel hierarchical ensemble methods is the incompleteness of the available annotations and its impact on the performance of computational methods for AFP. Indeed, the successful application of supervised and semisupervised machine learning methods to these tasks requires a gold standard for protein function, that is, a trusted set of correct examples, but unfortunately the annotations is incomplete and undergoes frequent updates, and also the GO is frequently updated. Some seminal works showed that, on the one hand, current machine learning approaches are able to generalize and predict novel biology from an incomplete gold standard and, on the other hand, incomplete functional annotations adversely affect the evaluation of machine learning performance [188]. A very recent work addressed these items by proposing methods based on* weak-label learning* specifically designed to replenish the functions of proteins under the assumption that proteins are partially annotated. More precisely, two new algorithms have been proposed:* ProWL*, protein function prediction with weak-label learning, which can recover missing annotations by using the available* relevant* annotations, that is, a set of trusted annotations for a given protein, and* ProWL-IF*, protein function prediction with weak-label learning and knowledge of irrelevant function, by which also* irrelevant* functions, that is, functions that cannot be associated with the protein of interest, are exploited to replenish the missing functions [189, 190]. The results show that these items should be considered in future works for hierarchical multilabel predictions of protein functions in model organisms.

Another issue is represented by the reliability of the annotations. Usually, only experimental evidence is used to annotate the proteins for training AFP methods, but most of the available annotations are computationally predicted annotations without any experimental validation [191]. To at least partially exploit this huge amount of information, computational methods able to take into account the different reliability of the available annotations should be developed and integrated into hierarchical ensemble algorithms.

A quite neglected item is the interpretability of the hierarchical models. Nevertheless, the generation of comprehensible classification models is of paramount importance for biologists in order to provide new insights into the correlation of protein features and their functions [192]. A first step in this direction is represented by the work of Cerri et al. that exploits the advantages of grammar-based evolutionary algorithms to incorporate prior knowledge with the simplicity of genetic algorithms for optimization problems in order to produce interpretable rules for hierarchical multilabel classification [193].

Other issues depend on “strength” or the general rule that relates the predictions made by the base learner at a given term/node of the hierarchy with the predictions made by the other base learners of the hierarchical ensemble. For instance, the TPR algorithm (Section 7) weights the positive predictions of deeper nodes with an exponential decrement with respect to their depth (Section 11), but other rules (e.g., linear or polynomial) could be considered as the basis for the development of new algorithms that put more weight on the decisions of deep nodes of the hierarchy. Other enhancements could be introduced with the TPR-W algorithm (Section 7.2); indeed, we can note that positive children of a node at level *i* of the hierarchy have the same weight, independently of the size of their hanging subtree. In some cases, this could be useful, but in other cases it could be desirable to directly take into account the fact that a positive prediction is maintained along a path of the tree; indeed, this witnesses for a positive annotation of the node at level *i*.

More in general, in the spirit of a work recently proposed [123], the analysis of the synergy between the issues introduced above could be of great interest to better understand the behaviour of hierarchical ensemble methods.

Finally, we introduce some problems that could open new and interesting research lines in the context of hierarchical ensemble methods.

At first, an important issue could be represented by the design and development of multitask learning strategies [194] able to exploit the relationships between functional classes just during the learning phase, in order to establish a functional connection between learning processes associated with hierarchically related classes of the functional taxonomy. In this way, just during the training of the base learners, the learning processes will be dependent on each other (at least for nearby nodes/classes), enabling “mutual learning” of related classes in the taxonomy.

A second, to my knowledge, not explored learning issue is represented by the metaintegration of hierarchical predictions. Considering that there is no “killer” hierarchical ensemble method, a metacombination of the hierarchical predictions could be explored to enhance the overall performances.

A last issue is represented by multispecies predictions in a hierarchical context. By exploiting homology relationships between proteins of different species, we could enhance the prediction for a particular species by using predictions or data available for other species. This is a common practice with, for example, sequence-based methods, but novel research is needed to extend this homology-based approach in the context of hierarchical ensemble methods for multispecies prediction. It is worth noting that this multispecies approach yields to big-data analysis with the associated problems of scalability of existing algorithms. A possible solution to this last problem could be represented by distributed parallel computation [195] or by the adoption of secondary memory-based computational techniques [196].

## 11. Conclusions

Hierarchical ensemble methods represent one of the main research lines for AFP. Their two-step learning strategy introduces a high modularity in the prediction system: in the first step, different base learners can be trained to individually learn the functional classes, and in the second step different algorithms can be chosen to hierarchically combine the predictions provided by the base classifiers. The best results can be obtained when the global topology of the ontology is exploited and when both top-down and bottom-up learning strategies are applied [24, 27, 30, 31].

Nevertheless, a hierarchical learning strategy alone is not enough to achieve state-of-the-art results for AFP. Indeed, we need to design hierarchical ensemble methods in the context of the learning issues strictly related to the AFP problem.

The first one is represented by data fusion, since each source of biomolecular data may provide different and often complementary information about a protein and an integration of data fusion methods with hierarchical ensembles is mandatory to improve AFP results.

The second one is represented by the cost-sensitive techniques needed to take into account the usually small number of positive annotations: data unbalance-aware methods should be embedded in hierarchical methods to avoid solutions biased toward low sensitivity predictions.

Other issues, ranging from the proper choice of negative examples to the reliability and the incompleteness of the available annotation, the balance between local and global learning strategies, and the metaintegration of hierarchical predictions have been only partially addressed in previous work. More in general, the synergy between hierarchical ensemble methods, data integration algorithms, cost-sensitive techniques, and other related issues is the key to improve AFP methods and to drive experiments aimed at discovering previously unannotated or partially annotated protein functions [123].

Indeed, despite their successful application to protein function prediction in different model organisms, as outlined in Section 9, there is large room for future research in this challenging area of computational biology.

In particular, the development of multitask learning methods to jointly learn related GO terms in a hierarchical context and the design of multispecies hierarchical algorithms, able to scale with millions of proteins, represent a compelling challenge for the computational biology and bioinformatics community.

## Figures and Tables

**Figure 16:**
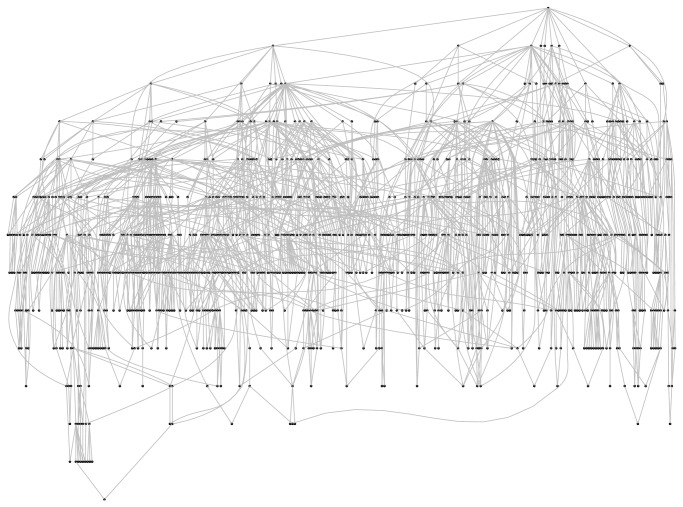
GO BP DAG for the yeast model organism (realized through the* HCGene* software [178]), involving more than 1000 terms and more than 2000 edges.

**Figure 17:**
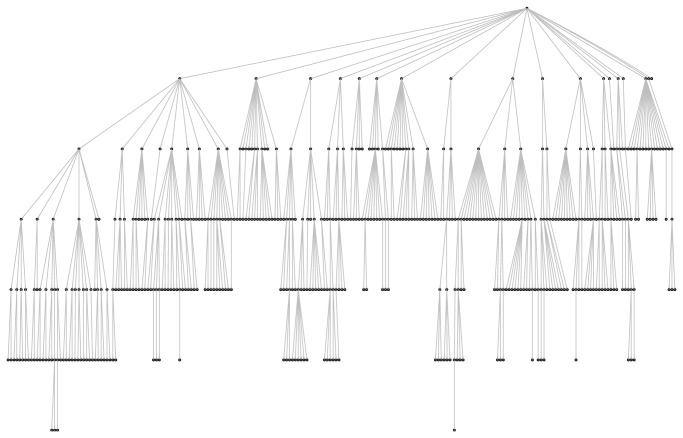
FunCat tree for the yeast model organism (realized through the* HCGene* software) [178]. A “dummy” root node has been added to obtain a single tree from the tree forest.

**Figure 18:**
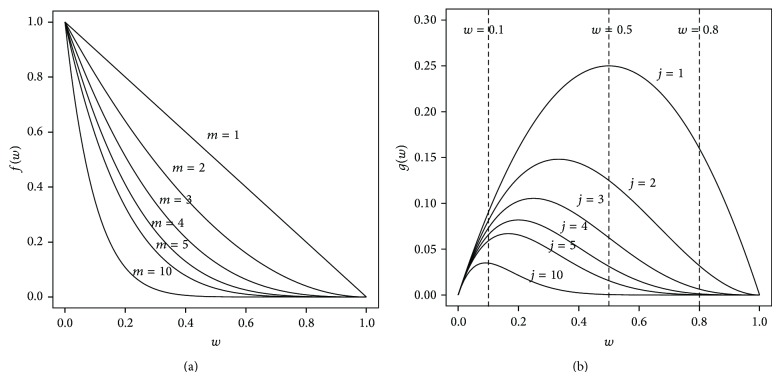
(a) Plot of *f*(*w*) = (1 − *w*)^*m*^, while varying *m* from 1 to 10. (b) Plot of *g*(*w*) = *w*(1 − *w*)^*j*^, while varying *j* from 1 to 10. The integers *j* refer to internal nodes at distance *j* from the reference node at level *k*.

**Table 1:** Characteristics of some of the main hierarchical ensemble methods for AFP.

Methods	References	Multipath	Partial path	Class structure	Cost sens.	Sel neg.	Base learn	Improves on flat	Node process
HMC-LMLP	[124, 125]	√	√	TREE			any	√	TD
HTD-CS	[27]	√	√	TREE	√		any	√	TD
HTD-MULTI	[127]		√	TREE			any	√	TD
HTD-PERLEV	[128]			TREE			spec	√	TD
HTD-NET	[26]	√	√	DAG			any	√	TD
BAYES NET-ENS	[20]	√	√	DAG		√	spec	√	TD & BUP
HIER-MB and BFS	[30]	√	√	DAG			any	√	TD & BUP
HBAYES	[92, 135]	√	√	TREE		√	any	√	TD & BUP
HBAYES-CS	[123]	√	√	TREE	√	√	any	√	TD & BUP
Reconc-heuristic	[24]	√	√	DAG			any	√	TD
Cascaded log	[24]	√	√	DAG			any	√	TD
Projection-based	[24]	√	√	DAG			any	√	TD & BUP
TPR	[31, 142]	√	√	TREE		√	any	√	TD & BUP
TPR-W	[31]	√	√	TREE	√	√	any	√	TD & BUP
TPR-W weighted	[145]	√	√	TREE	√		any	√	TD & BUP
Decision-tree-ens	[29]	√	√	DAG			spec	√	TD & BUP

**Table 2:** Comparison of the results (average per class *F*-scores) achieved with single sources and multisource (data fusion) techniques. FLAT, HTD, HTD-CS, HB (HBAYES), HB-CS (HBAYES-CS), TPR, and TPR-W ensemble methods are compared with and without data integration. In the last row, the number in parentheses refers to the percentage relative increment in *F*-score performance achieved with data fusion techniques with respect to the best single source of evidence (BIOGRID).

Methods	FLAT	HTD	HTD-CS	HB	HB-CS	TPR	TPR-W
Single-source							
BIOGRID	0.2643	0.3759	0.4160	0.3385	0.4183	0.3902	0.4367
String	0.2203	0.2677	0.3135	0.2138	0.3007	0.2801	0.3048
PFAM BINARY	0.1756	0.2003	0.2482	0.1468	0.2407	0.2532	0.2738
PFAM LOGE	0.2044	0.1567	0.2541	0.0997	0.2847	0.3005	0.3160
Expr.	0.1884	0.2506	0.2889	0.2006	0.2781	0.2723	0.3053
Seq. sim.	0.1870	0.2532	0.2899	0.2017	0.2825	0.2742	0.3088

Multisource (data fusion)							
Kernel fusion	0.3220(22)	0.5401(44)	0.5492(32)	0.5181(53)	0.5505(32)	0.5034(29)	0.5592(28)

## Appendices

### A. Gene Ontology and FunCat

The two main taxonomies of gene functional classes are represented by the Gene Ontology (GO) [6] and the functional catalogue (FunCat) [5]. In the former, the functional classes are structured according to a directed acyclic graph, that is, a DAG (Figure 16), while in the latter, the functional classes are structured through a forest of trees (Figure 17). The GO is composed of thousands of functional classes, and it is set out in three separated ontologies: “biological processes,” “molecular function,” and “cellular component”. Indeed, a gene can participate to specific biological processes (e.g., cell cycle, metabolism, and nucleotide biosynthesis) and at the same time can perform specific molecular functions (e.g., catalytic or binding activities that occur at the molecular level) in specific cellular components (e.g., mitochondrion or rough endoplasmic reticulum).

#### A.1. GO: The Gene Ontology

The Gene Ontology (GO) project began as collaboration between three model organism databases, FlyBase (*Drosophila*), the* Saccharomyces* genome database (SGD), and the mouse genome database (MGD), in 1998. Now, it includes several of the world's major repositories for plant, animal, and microbial genomes. The GO project has developed three structured controlled vocabularies (ontologies) that describe gene products in terms of their associated biological processes, cellular components, and molecular functions in a species-independent manner (Figure 16). Biological process (BP) represents series of events accomplished by one or more ordered assemblies of molecular functions that exploit a specific biological function. for instance, lipid metabolic process or tricarboxylic acid cycle. Molecular function (MF) describes activities that occur at the molecular level, such as catalytic or binding activities. An example of MF is glycine dehydrogenase activity or glucose transporter activity. Cellular component (CC) represents just parts or components of a cell, such as organelles or physical places or compartments in which a specific gene product is located. An example is the endoplasmic reticulum or the ribosome.

The ontologies of the GO are structured as a directed acyclic graph (DAG) *G* = 〈*V*, *E*〉, where *V* = {*t*∣terms  of  the  GO} and *E* = {(*t*, *u*)∣*t*, *u* ∈ *V*}. Relations between GO terms are also categorized in the following three main groups:

- *is-a* (subtype relations): if we say the term/node *A* is a *B*, we mean that node *A* is a subtype of node *B*. For example, mitotic cell cycle is a cell cycle, or lyase activity is a catalytic activity;
- *part-of* (part-whole relations): *A* is part of *B* which means that whenever *A* exists, it is part of *B*, and the presence of *A* implies the presence of *B*. For instance, mitochondrion is part of cytoplasm;
- *regulates* (control relations): if we say that *A* regulates *B* we mean that *A* directly affects the manifestation of *B*; that is, the former regulates the latter.

While* is-a* and* part-of* are transitive, “*regulates*” is not. Moreover, in some cases, regulatory proteins are not expected to have the same properties as the proteins they regulate and hence predicting regulation may require other data and assumptions than predicting function similarity. For these reasons, usually in AFP,* regulates* relations (that however are a minority of the existing relations) are usually not used.

Each annotation is labeled with an* evidence code* that indicates how the annotation to a particular term is supported. They are subdivided in several categories ranging from experimental evidence codes, used when experimental assays have been applied for the annotation, for example, inferred from physical interaction (IPI) or inferred from mutant phenotype (IMP) or inferred from genetic interaction (IGI), to author statement codes, such as traceable author statement (TAS), that indicate that the annotation was made on the basis of a statement made by the author(s) in the cited reference, to computational analysis evidence codes, based on an in silico analyses manually reviewed (e.g., inferred from sequence or structural similarity (ISS)). For the full set of available evidence codes, please see the GO website (http://www.geneontology.org/).

A GO graph for the yeast model organism is represented in Figure 16. It is worth noting that, despite the complexity of the represented graph, Figure 16 does not show all the available terms and the relationships involved in the GO BP ontology with the yeast model organism.

#### A.2. FunCat: The Functional Catalogue

The FunCat taxonomy started with* Saccharomyces cerevisiae* genome project at MIPS (http://mips.gsf.de/): at the beginning, FunCat contained only those categories required to describe yeast biology [197, 198], but successively its content has been extended to plants to annotate genes from the* Arabidopsis thaliana* genome project and furthermore to cover prokaryotic organisms and finally animals too [5].

The* FunCat* represents a relatively simple and concise set of gene functional classes: it consists of 28 main functional categories (or branches) that cover general fields like cellular transport, metabolism, and cellular communication/signal transduction. These main functional classes are divided into a set of subclasses with up to six levels of increasing specificity, according to a tree-like structure that accounts for different functional characteristics of genes and gene products. Genes may belong at the same time to multiple functional classes, since several classes are subclasses of more general ones, and because a gene may participate in different biological processes and may perform different biological functions.

Taking into account the broad and highly diverse spectrum of known protein functions, the FunCat annotation scheme covers general features, like cellular transport, metabolism, and protein activity regulation. Each of its main 28 functional branches is organized as a hierarchical, tree-like structure, thus leading to a tree forest with hundreds of functional categories.

Differently from the GO, the FunCat is more compact and does not intend to classify protein functions down to the most specific level. From a general standpoint, it can be compared to parts of the molecular function and biological process terms of the GO system.

One of the main advantages of FunCat is its intuitive category structure. For instance, the annotation of yeast uses only 18 of the main categories and less than 300 distinct categories (Figure 17), while the* Saccharomyces* genome database (SGD) [199] uses more than 1500 GO terms in its yeast annotation. Indeed, FunCat focuses on the functional process and in part on the molecular function, while GO aims at representing a fine granular description of proteins that provides annotations with a wealth of detailed information. However, to achieve this goal, the detailed description offered by GO leads to a large number of terms (e.g., the ontology for biological processes alone contains more than 10000 terms), and such a huge amount of terms is very difficult to be handled for annotators. Moreover, we may have a very large number of possible assignments that may lead to erroneous or inconsistent annotations. FunCat is simpler: its tree structure, compared with the DAG structure of the GO, leads to both simple procedures for annotation and less difficult computational-based classification tasks. In other words, it represents a well-balanced compromise between extensive depth, breadth, and resolution but without being too granular and specific.

It is worth noting that both FunCat and GO ontologies undergo modifications between different releases, and at the same time the annotations are also subjected to changes, since they represent the results of the knowledge of the scientific community at a given time. As a consequence, predictions resulting, for example, in false positives for a given release of the GO, may become true positive in future releases, and more in general we should keep in mind that the available annotations are always partial and incomplete and depend on the knowledge available for the species under study. Nevertheless, even if some works pointed out the inconsistency of current GO taxonomies through the analysis of violations of terms univocality [200], GO and FunCat are considered the ground truth to evaluate AFP methods, since they represent the main effort of the scientific community to organize commonly accepted taxonomy of protein functions [191].

### B. AFP Performance Assessment in a Hierarchical Context

In the context of ontology-wide protein function prediction problems, where negative examples are usually a lot more than positive ones, accuracy is not a reliable measure to assess the classification performance. For this reason, the classical* F*-score is used instead, to take into account the unbalance of functional classes. If TP represents the positive examples correctly predicted as positive, FN, the positive examples incorrectly predicted as negative, and, FP, the negatives incorrectly predicted as positives, then the precision *P*, and the recall *R* are

(B.1) $$\begin{array}{c} P=\frac{\text{TP}}{\text{TP}+\text{FP}}\;\;R=\frac{\text{TP}}{\text{TP}+\text{FN}}. \end{array}$$

The* F*-score *F* is the harmonic mean between precision and recall

(B.2) $$\begin{array}{c} F=\frac{2\cdot P\cdot R}{P+R}. \end{array}$$

If we need to evaluate the correct ranking of annotated proteins with respect to a specific functional class, a valuable measure is represented by the area under the receiving operating characteristic curve (AUC). A random ranking corresponds to AUC≃0.5, while values close to 1 correspond to near optimal ranking; that is, AUC = 1, if all the annotated genes are ranked before the unannotated ones.

In order to better capture the hierarchical and sparse nature of the protein function prediction problem, we also need specific measures that estimate how far a predicted structured annotation is from the correct one. Indeed, functional classes are structured according to a direct acyclic graph (Gene Ontology) or to a tree (FunCat), and we need measures to accommodate not just “exact matches” but also “near misses” of different sorts.

For instance, correctly predicting a parent or ancestor annotation, while failing to predict the most specific available annotation, should be “partially correct,” in the sense that we can gain information about the more general functional characteristics of a gene, missing only its most specific functions.

More precisely, given a general taxonomy *G* representing the graph of the functional classes, for a given gene/gene product *x*, consider the graph *P*(*x*) ⊂ *G* of the predicted classes and the graph *C*(*x*) of the correct classes associated with *x*, and let *l*(*P*) be the set of the leaves (nodes without children) of the graph *P*. Given a leaf *p* ∈ *P*(*x*), let ↑*p* be the set of ancestors of the node *p* that belong to *P*(*x*), and, given a leaf *c* ∈ *C*(*x*), let ↑*c* be the set of ancestors of the node *c* that belong to *C*(*x*); the hierarchical precision (HP), hierarchical recall (HR), and hierarchical *F*-score (HF) are defined as follows [201]:

(B.3) $$\begin{array}{c} \text{HP}=\frac{1}{\left|l\left(P\left(x\right)\right)\right|}\underset{p\in l(P(x))}{\sum }\underset{c\in l(C(x))}{\max}\frac{\left|↑c\cap ↑p\right|}{\left|↑p\right|}\\ \text{HR}=\frac{1}{\left|l\left(C\left(x\right)\right)\right|}\underset{c\in l(C(x))}{\sum }\underset{p\in l(P(x))}{\max}\frac{\left|↑c\cap ↑p\right|}{\left|↑c\right|}\\ \text{HF}=\frac{2\cdot \text{HP}\cdot \text{HR}}{\text{HP}+\text{HR}}. \end{array}$$

In the case of the FunCat taxonomy, since it is structured as a tree, we can simplify HP, HR, and HF as follows:

(B.4) $$\begin{array}{c} \text{HP}=\frac{1}{\left|l\left(P\left(x\right)\right)\right|}\underset{p\in l(P(x))}{\sum }\frac{\left|C\left(x\right)\cap ↑p\right|}{\left|↑p\right|}\\ \text{HR}=\frac{1}{\left|l\left(C\left(x\right)\right)\right|}\underset{c\in l(C(x))}{\sum }\frac{\left|↑c\cap P\left(x\right)\right|}{\left|↑c\right|}\\ \text{HF}=\frac{2\cdot \text{HP}\cdot \text{HR}}{\text{HP}+\text{HR}}. \end{array}$$

An overall high hierarchical precision is indicating that the predictor is able to detect the most general functions of genes/gene products. On the other hand, a high average hierarchical recall indicates that the predictors are able to detect the most specific functions of the genes. The hierarchical* F*-measure expresses the correctness of the structured prediction of the functional classes, taking into account also partially correct paths in the overall hierarchical taxonomy, thus providing in a synthetic way the effectiveness of the structured hierarchical prediction.

Another variant of hierarchical classification measure is represented by the hierarchical* F*-measure proposed by Kiritchenko et al. [202]. Let *P*(*x*) be the set of classes predicted in the overall hierarchy for a given gene/gene product *x*, and let *C*(*x*) be the corresponding set of “true” classes. Then, the hierarchical precision HP_*K*_ and the hierarchical recall HR_*K*_ according to Kiritchenko are defined as

(B.5) $$\begin{array}{c} \text{H}{\text{P}}_{K}=\underset{x}{\sum }\frac{\left|P\left(x\right)\cap C\left(x\right)\right|}{\left|P\left(x\right)\right|}\;\;\text{H}{\text{R}}_{K}=\underset{x}{\sum }\frac{\left|P\left(x\right)\cap C\left(x\right)\right|}{\left|C\left(x\right)\right|}. \end{array}$$

Note that these definitions do not explicitly consider the paths included in the predicted subgraphs but simply the ratio between the number of common classes and, respectively, the predicted (HP_*K*_) and the true classes (HR_*K*_). The hierarchical* F*-measure HF_*K*_ is the harmonic mean between HP_*K*_ and HR_*K*_

(B.6) $$\begin{array}{c} \text{H}{\text{F}}_{K}=\frac{2\cdot \text{H}{\text{P}}_{K}\cdot \text{H}{\text{R}}_{K}}{\text{H}{\text{P}}_{K}+\text{H}{\text{R}}_{K}}. \end{array}$$

### C. Effect of the Propagation of the Positive Decisions in TPR Ensembles

In TPR ensembles, a* generic* node at level *k* is any node whose distance from the root is equal to *k*. The posterior probability computed by the ensemble for a generic node at level *k* is denoted by *q*_*k*_. More precisely, *q*_*k*_ denotes the probability computed by the ensemble and ${\hat{q}}_{k}$ denotes the probability computed by the base learner local to a node at level *k*. Moreover, we define *q*_*k*+1_^*j*^ as the probability of a child *j* of a node at level *k*, where the index *j* ≥ 1 refers to different children of a node at level *k*. From (30), we can derive the following expression for the probability *q*_*k*_ computed for a generic node at level *k* of the hierarchy [31]:

(C.1) $$\begin{array}{c} q_{k}\left(g\right)=w\cdot {\hat{q}}_{k}\left(g\right)+\frac{1-w}{\left|\phi_{k}\left(g\right)\right|}\underset{j\in \phi_{k}\left(g\right)}{\sum }q_{k+1}^{j}\left(g\right). \end{array}$$

To simplify the notation, we can introduce the following expression to indicate the average of the probabilities computed by the positive children nodes of a generic node at level *k*:

(C.2) $$\begin{array}{c} a_{k+1}=\frac{1}{\left|\phi_{k}\right|}\underset{j\in \phi_{k}}{\sum }{\hat{q}}_{k+1}^{j} \end{array}$$

and we can introduce similar notations for *a*_*k*+2_ (average of the probabilities of the grandchildren) and more in general for *a*_*k*+*j*_ (descendants at level *j* of a generic node). By extending these definitions across levels, we can obtain the following theorem.

Theorem 2 (influence of positive descendant nodes). In a TPR-W ensemble, for a generic node at level *k*, with a given parameter *w*, 0 ≤ *w* ≤ 1, balancing the weight between parent and children predictors, and having a variable number larger than or equal to 1 of positive descendants for each of the *m* lower levels below, the following equality holds for each *m* ≥ 1:

(C.3) $$\begin{array}{c} q_{k}=w{\hat{q}}_{k}+\overset{m-1}{\underset{j=1}{\sum }}w{\left(1-w\right)}^{j}a_{k+j}+{\left(1-w\right)}^{m}a_{k+m}. \end{array}$$

For the full proof, see [31].

Theorem 2 shows that the contribution of the descendant nodes decays exponentially with their depth and depends critically on the choice of the *w* parameter. To get more insights into the relationships between *w* and its effect on the influence of positive decisions on a generic node at level *k* (Theorem 1), Figure 18(a) shows the function that governs the decay of the influence of leaf nodes at different depths *m*, and Figure 18(b) shows the function responsible of the influence of nodes above the leaves.
